# CRISPR/Cas9-Mediated Mutation in *XSP10* and *SlSAMT* Genes Impart Genetic Tolerance to Fusarium Wilt Disease of Tomato (*Solanum lycopersicum* L.)

**DOI:** 10.3390/genes14020488

**Published:** 2023-02-14

**Authors:** Johni Debbarma, Banashree Saikia, Dhanawantari L. Singha, Debajit Das, Ajay Kumar Keot, Jitendra Maharana, Natarajan Velmurugan, Kallare P. Arunkumar, Palakolanu Sudhakar Reddy, Channakeshavaiah Chikkaputtaiah

**Affiliations:** 1 Biological Sciences and Technology Division, CSIR-North East Institute of Science and Technology (CSIR-NEIST), Jorhat 785006, Assam, India; 2 Academy of Scientific and Innovative Research (AcSIR), Ghaziabad 201002, Uttar Pradesh, India; 3 Department of Agricultural Biotechnology, Assam Agricultural University, Jorhat 785013, Assam, India; 4 Branch Laboratory-Itanagar, Biological Sciences Division, CSIR-NEIST, Naharlagun 791110, Arunachal Pradesh, India; 5 Central Muga Eri Research and Training Institute (CMER&TI), Lahdoigarh, Jorhat 785700, Assam, India; 6 International Crop Research Institute for the Semi Arid Tropics (ICRISAT), Hyderabad 502324, Telangana, India

**Keywords:** CRISPR/Cas9, dual-gene editing, INDEL, Fusarium wilt, genetic tolerance, tomato

## Abstract

Fusarium wilt is a major devastating fungal disease of tomato (*Solanum lycopersicum* L.) caused by *Fusarium oxysporum* f. sp. *lycopersici* (*Fol*) which reduces the yield and production. Xylem sap protein 10 (XSP10) and Salicylic acid methyl transferase (SlSAMT) are two putative negative regulatory genes associated with Fusarium wilt of tomato. Fusarium wilt tolerance in tomato can be developed by targeting these susceptible (*S*) genes. Due to its efficiency, high target specificity, and versatility, CRISPR/Cas9 has emerged as one of the most promising techniques for knocking out disease susceptibility genes in a variety of model and agricultural plants to increase tolerance/resistance to various plant diseases in recent years. Though alternative methods, like RNAi, have been attempted to knock down these two *S* genes in order to confer resistance in tomato against Fusarium wilt, there has been no report of employing the CRISPR/Cas9 system for this specific intent. In this study, we provide a comprehensive downstream analysis of the two *S* genes via CRISPR/Cas9-mediated editing of single (*XSP10* and *SlSAMT* individually) and dual-gene (*XSP10* and *SlSAMT* simultaneously). Prior to directly advancing on to the generation of stable lines, the editing efficacy of the sgRNA-Cas9 complex was first validated using single cell (protoplast) transformation. In the transient leaf disc assay, the dual-gene editing showed strong phenotypic tolerance to Fusarium wilt disease with INDEL mutations than single-gene editing. In stable genetic transformation of tomato at the GE_1_ generation, dual-gene CRISPR transformants of *XSP10* and *SlSAMT* primarily exhibited INDEL mutations than single-gene-edited lines. The dual-gene CRISPR-edited lines (CRELs) of *XSP10* and *SlSAMT* at GE_1_ generation conferred a strong phenotypic tolerance to Fusarium wilt disease compared to single-gene-edited lines. Taken together, the reverse genetic studies in transient and stable lines of tomato revealed that, *XSP10* and *SlSAMT* function together as negative regulators in conferring genetic tolerance to Fusarium wilt disease.

## 1. Introduction

*Fusarium oxysporum* f. sp. *lycopersici* (*Fol*) is a fungal pathogen that infects the roots of plants [1] and is ranked as the fifth most devastating fungal infection in tomato [2]. *Fol* hyphae penetrate and colonize the apoplastic spaces, encircling the stele and clogging xylem vessels, causing slower growth, chlorosis of the leaves, progressive wilting, and cell death [3,4]. Tomato (*Solanum lycopersicum* L.) is widely cultivated across the globe for consumption and processed products [5]. In India, the disease incidence of Fusarium wilt in tomato ranges from 25–55%, with production losses reaching up to 80% under conducive environmental conditions [6]. During *Fol* infection, the host’s fusarium wilt disease susceptibility genes (*S*) greatly increase infection and enhance compatibility [7]. Additionally, the “*S*” genes are vital in meeting the pathogen’s metabolic or structural requirements, contributing to sustenance and pathogen proliferation [7]. They essentially work as negative regulators, suppressing plant host defense responses.

Xylem sap protein 10 (XSP10) is a 10 kDa non-specific lipid transfer protein (LTPs) with an 8-cysteine residue motif that creates intramolecular disulfide linkages in tomato [8]. According to studies, the *XSP10* gene acts as a compatibility factor for *Fol*, enhancing *Fol* colonization in the tomato plant’s root and contributing to the development of disease symptoms in the plants [9]. In addition, it transfers essential lipids from the intracellular membrane to pathogens, increasing disease susceptibility and progression [10]. Apparently, *XSP10* is highly expressed in roots and moderately in stems of tomato [8]. In tomato, approximately 54% of the reported xylem sap proteins are found in exosomes [4], but in cotton, it is found in the apoplastic space [11].

Another *S* gene of tomato is the salicylic acid methyl transferase (*SlSAMT*), which belongs to the class of O-methyl transferases. SAMT enzymes regulate the salicylic acid (SA) homeostasis within the plant by catalyzing the conversion of salicylic acid (SA) to methyl salicylate (MeSA) with *S*-adenosyl-l-methionine (SAM) as a methyl donor [12]. The conversion of endogenous SA to MeSA reduces the host’s defense against multiple pathogen attacks [13]. Additionally, systemic acquired resistance (SAR) is reliant on salicylic acid (SA) to induce the plant defense arsenal against a broad spectrum of pathogens [14]. Therefore, the activity of *SlSAMT* negatively regulates the SAR against pathogen infection. On the other hand, silencing of *SlSAMT* through RNAi reduced susceptibility to virulent fungal pathogen *Fol* in tomato [13], whereas overexpression of *OsBSMT1* and *AtBSMT1* exhibited MeSA productions and resulted in disease susceptibility to the pathogens *Golovinomyces orontii* and *Pseudomonas syringae* in rice (*Oryza sativa* L.) and Arabidopsis (*Arabidopsis thaliana*), respectively [14,15]. 

*Xylem sap protein 10* (*XSP10*) and *salicylic acid methyle transferase* (*SlSAMT*) have been identified as putative negative regulatory genes associated to Fusarium wilt disease of tomato. Despite their importance as potential candidate genes, very little knowledge is known about their genetic tolerance to *Fol*. To date, most of the crops’ resistance genes to Fusarium wilt have been inadequately targeted by introgression breeding [16]. Therefore, an integrated approach assimilating genome editing techniques is deemed necessary to curtail plant disease hypersensitivity [17]. Targeted genome editing has emerged as an alternative to conventional breeding and genetic engineering methods for sustainable food production [18] and disease resistance [19]. Clustered regularly interspaced short palindromic repeats (CRISPR)/CRISPR-associated protein (CRISPR/Cas9) and 20-bp guide RNA form a complex, induce cleavage at target specific genomic loci, and facilitate mutation in plants [20]. Due to its simplicity, robustness, target specificity, minimal off-target effects, and non-tedious nature, CRISPR/Cas9 tools are promising compared to zinc finger nucleases (ZFNs) and transcription activator-like effector endonuclease (TALENs) [21]. The CRISPR/Cas9-based genome editing system has been successfully used in tobacco [22], wheat [23], potato, soybean [24], rice, and maize [25] to knock-out of different negative regulatory genes. 

The current work sought to leverage CRISPR/Cas9 technology to precisely edit two key negative regulatory genes, *XSP10* and *SlSAMT*, that weaken the defensive response of tomato cultivar Arka Vikas (cv. AV) to Fusarium wilt disease, using the multiple disease resistant cultivar Arka Abhed (cv. AA) as a control check. Among the S genes, we chose *XSP10* and *SlSAMT* for the Fusarium wilt disease tolerance study mainly based on previous studies [9,13] and the findings from our most recent article [26], in which we showed the differential expression of *XSP10* and *SlSAMT* in cv. AV and cv. AA as well as their strong protein–protein interaction through in silico and in vivo Bimolecular Fluorescence Complementation (BiFC) analysis. Although there are reports of alternative reverse genetics approaches, CRISPR/Cas9-mediated targeted editing of these two “*S*” genes for conferring Fusarium wilt resistance in tomato has not yet been documented. Our results exemplified that CRISPR editing of *XSP10* and *SlSAMT* conferred Fusarium wilt tolerance in tomato cv. AV by restricting fungal colonization in the root, curbing ROS over-accumulation, and manifestation of disease symptoms.

## 2. Materials and Methods

### 2.1. Plant Materials and Growth Condition 

The seeds of *S. lycopersicum* L. (Arka Vikas cultivar) were obtained from the Indian Institute of Horticulture Research (IIHR, Bangalore, India). The seeds were surface-sterilized for 5 min with 70% ethanol and seeds were resuspended in 4% sodium hypochlorite (*v*/*v*) for 10 min before being rinsed three times with sterile distilled water. The sterile seeds were grown for 10–12 days in half-strength MS (Murashige and Skoog) media supplemented with 3% sucrose and 0.3% Gelrite (Sigma-Aldrich, St. Louis, MO, USA) at 25–28 °C and 70% relative humidity (RH) in a plant growth chamber with a 16/8 h light-dark photoperiod. Hypocotyls that were well-grown and healthy were used in the experiment.

### 2.2. sgRNA Design and CRISPR/Cas9 Construct Generation

The 19-bp nucleotide sgRNA was designed for *XSP10* (TGAGAATGCATCCGTATCA) on the first exonic region and for *SlSAMT* (TTCACTTCAATGATCTCCC) on the second exonic region (upstream of PAM site), taking several parameters into account, such as GC content (50–55%), specificity and efficiency (50–100%), minimum off-targets (4-bp mismatch), and secondary structure (6-bp) using online bioinformatics tools, namely CCTop (https://cctop.cos.uni-heidelberg.de:8043/) accessed on 10 April 2020 [27] and CHOPCHOP (https://chopchop.cbu.uib.no/) accessed on 10 April 2020 [28]. To generate annealed oligonucleotides in a PCR thermocycler, the designed 19-nucleotide protospacer of forward (ATTG site) and reverse primers (AAAC site) with 4 bp overhang were diluted to a final concentration of 10 μM, heated first at 98 °C for 10 s, followed by 55 °C for 10 min, and slowly cooled to 25 °C. The pFH6 vector backbone (gRNA entry Vector) of 3612 bp was linearized with BbsI-HF enzyme. The oligo-annealed protospacer was inserted into the linear pFH6 vector between the *U6 promoter* and sgRNA scaffold by T4 DNA ligase (Invitrogen, Thermo Fisher SCIENTIFIC, Life Technologies, Carlsbad, CA, USA). The ligated product was transformed into DH5α competent cells and incubated for 2 h at 37 °C, followed by selection of the transformed colonies in LB medium supplemented with ampicillin (100 mg/L). The sgRNA cassettes from the positive clones were first amplified by PCR with amplicons size of 416 bp followed by gel extraction and purification using a mini elute Gel extraction kit (Cat No: 28604, Qiagen, Hilden, Germany). The insertion of sgRNA was confirmed by Sanger sequencing of the purified PCR product. The sgRNA entry clone was assembled into Cas9 expression vector p63 plasmid using the Gibson assembly method [29], and positive clones were confirmed by digestion with EcoRI and HindIII restriction enzymes (Invitrogen, Thermo Fisher SCIENTIFIC, Life Technologies). 

### 2.3. Protoplast Transfection and Genomic DNA Isolation of CRISPR/Cas9 Constructs

About 10 μg of CRISPR/Cas9 binary constructs of *XSP10* and *SlSAMT* were transformed into 200 μL of tomato protoplast cells through the PEG method [30]. The transfected cells were incubated for 48 h at room temperature under darkness. Single-gene CRISPR/Cas9 constructs are abbreviated as *XSP10* (SX) and *SlSAMT* (SS), while dual-gene constructs are abbreviated as *XSP10*-*SlSAMT* (DXS). CRISPR/Cas9 constructs of SX and SS were independently transformed into protoplasts for single-gene editing analysis, while CRISPR/Cas9 constructs of SX and SS were co-transformed (DXS) for dual-gene editing. Next, the genomic DNA from all three sets of CRISPR/Cas9 transformed protoplasts was extracted using the sodium dodecyl sulfate (SDS) method [31]. 

### 2.4. Agrobacterium-Infiltration in Tomato Fruit and Leaves

The p63 CRISPR/Cas9 expression plasmids harboring the single guide RNA (sgRNA) of *XSP10* and *SlSAMT* were transformed into the *Agrobacterium tumefaciens* LBA4404 strain following the electroporation method [32]. For generating dual-gene (DXS) constructs, SX and SS Agrobacterial suspension cell culture was mixed gently. Bacterial culture was streaked and grown in 50 mL YEB suspension medium (beef extract 5 g/L, yeast extract 1 g/L, peptone 5 g/L, sucrose 5 g/L, and 0.5 g/L MgCl_2_ at pH 7) containing antibiotics 20 mg/L rifampicin, 100 mg/L streptomycin, and 100 mg/L kanamycin and incubated overnight at 28 °C. The OD_600_ was adjusted to 0.8. Then, 20 mL of bacterial culture was centrifuged at 5000 rpm for 10 min at 20 °C. The pellets were resuspended in an infiltration buffer (10 mM MgCl_2_, 10 mM MES at pH 5.6 and 100 µM Acetosyringone) and incubated for 30 min at 28 °C. 

For carrying out the β-glucuronidase (GUS) assay, ripened fruits of AV were collected from the greenhouse. About 200 μL of Agrobacterial suspension culture of single (SX and SS) and dual-gene (DXS) constructs was slowly injected into the stylar apex of fruit tissue separately by following the previously reported protocol [33]. 

A 1 mL syringe with a 0.65 × 60 mm needle was used to pierce the fruit tissues. For negative control, the fruits were injected with sterile water. Finally, the infiltrated fruits were kept at 25 °C for 2 days before GUS staining. 

For proper infiltration of Agrobacterium suspension of CRISPR/Cas9 constructs of single (SX and SS) and dual-gene DXS in leaves, leaf veins were pierced by a needle and small holes were created. About 3–5 leaves were taken and their abaxial sides were gently dipped in 30 mL of bacterial suspension culture for 20 min inside the desiccator with a vacuum pressure given at −22 in. Hg [34]. Leaves infiltrated with sterile water were taken as a negative control. The leaf discs were removed from the suspension culture and dried on sterile Whatman filter paper. Finally, leaf discs were incubated in a growth chamber at 25 °C with a 12 h:12 h light-dark cycle for 2 days with a light intensity of 60 μmol m^−2^ s^−1^ prior to GUS staining and pathogen (Fusarium) leaf detached assay.

### 2.5. Molecular Analysis of CRISPR-Editing Events in Protoplast and Leaves

To detect Cas9-induced mutagenesis in the tomato genome, PCR pre-screening was carried out using the Cas9 set of primers for each gene for single and dual-gene transformants. The wild-type genomic DNA isolated from untransformed protoplast cells was used as a negative control. The PCR profiling program was as follows: initial denaturation step at 94 °C for 5 min, followed by 35 cycles at 94 °C for 1 min, 56 °C for 45 sec and 72 °C for 30 sec, and then a final extension step at 72 °C for 7 min. The list of primers used for this experiment is given in Supplementary Table S1. PCR amplification of single-gene (SX and SS) and dual-gene (DXS) transformant cells was performed using gene-specific primers with Emerald Amp ^®^ GT PCR Master Mix (DSS-Takara, Cat. # RR310A). The amplicons were PCR purified and sub-cloned into PCR.2.1 (TA sub-cloning vector, Invitrogen™, Thermo Fisher SCIENTIFIC, Cat.# K202020) and about four positive clones from single-gene (SX and SS) and dual-gene (DXS) CRISPR/Cas9 transformants were sequenced using the Sanger method. The sequencing reads were then aligned with the reference gene and CRISPR/Cas9 editing events were analyzed using the Vector NTI software tool (Thermo Fisher, Life Technologies). Similarly, the presence of Cas9 from the genome of agro-infiltrated leaves of single (SX and SS) and dual-gene (DXS) was confirmed by PCR analysis. The PCR products were purified using the QIAquick^®^ PCR purification kit (Cat. No.28104), and the editing events were examined by Sanger sequencing. 

### 2.6. Stable Agrobacterium-Mediated Transformation of Tomato with the Binary CRISPR/Cas9 Constructs

Fusarium wilt susceptible tomato cv. AV was selected for stable lines Agrobacterium-mediated transformations following the protocol with little modification [35]. The hypocotyl segments of 10–12-day-old seedlings were co-transformed with *A. tumefaciens* (strain: LBA4404) harboring the binary CRISPR/Cas9 constructs of *XSP10* and *SlSAMT*. After the incubation of explants in Agrobacterium cell suspension culture for 15 min, hypocotyls were blot dried in sterile Whatman filter paper and shifted to pre-culture media, kept for 2 days at 25 °C in the dark condition. Next, the explants were sub-cultured to shooting media (MS media + sucrose + 1 mg/L of zeatin) bi-weekly for 1 month. The plates were normally kept at 25 °C, 70% RH, under a 16 h:8 h light-dark photoperiod. When shoots reached a minimum size of 1.5 cm, they were transferred to a selective rooting medium (SRM), and well-rooted plants were acclimatized in plastic bags containing a 4:2:2 mixture of cocopeat, vermiculite, and perlite. 

### 2.7. Genetic Analysis of CRISPR/Cas9 Editing Events and Off-Targets 

To confirm the presence of Cas9 in stable single and dual-gene CRISPR-edited lines (CRELs), the sodium dodecyl sulfate (SDS) method [31] was used to isolate genomic DNA from leaves of 3-week-old acclimatized putative plants. The Cas9 integration in the putative CRELs was confirmed with Cas9 primers, and Cas9 positive lines were PCR amplified using gene-specific primers (Supplementary Table S1). Then, PCR purified products were sent for Sanger sequencing (Bioserve Biotechnologies, India, Pvt. Ltd., Hyderabad, India). The CRISPR/Cas9 editing events were evaluated using online DSDecode M software (http://skl.scau.edu.cn/dsdecode/) accessed on 10 April 2020. Genotypic patterns such as homozygous, bi-allelic, and heterozygous were analyzed from superimposed sequencing chromatograms of sequenced PCR products in GE_0_ and GE_1_ lines [36]. However, DSDecode M software cannot decode sequencing chromatograms of complicated chimerics with more than 2–4 editing events [37]. Thus, those plants that could not be decoded were analyzed using ICE-Synthego software (https://ice.synthego.com/#/ accessed on 10 April 2020). Additionally, the sequencing chromatograms were manually checked for each edited line for appropriate data analysis [38].

The potential off-target sites were predicted with the CHOP-CHOP software (https://chopchop.cbu.uib.no/ accessed on 10 April 2020), as given in Supplementary Table S2. The primers were designed to flank the possible off-target sequences using Vector NTI software (Life-technologies). The PCR products were subjected to Sanger sequencing and aligned with the wild-type reference using DNAMAN.10 software (https://www.lynnon.com/ accessed on 10 April 2020). The genome sequences of wild-type (WT) and CRELs were evaluated critically to detect mutations in the loci other than the target sites. 

### 2.8. Histochemical GUS Staining

For histochemical ß-glucuronidase (GUS) analysis, 0.1 M 5-bromo-4-chloro-3-indolyl-β-D-glucuronide (X-Gluc) was used as a substrate and mixed with 1 M phosphate buffer (pH of 7.0), 0.5 M EDTA (pH-8), 50 mM potassium ferricyanide {K_3_Fe (CN)_6_}, 10% triton-X, and 20% methanol [39,40]. The tissues were thoroughly rinsed with sterile water before staining and incubated in the GUS stain solution at 37 °C. After 2 days, a blue color appeared on the surface of the tissues. To remove excess stain and chlorophyll content, 70% ethanol was used. Finally, the tissues were observed under light microscopy (Leica Microsystems, Wetzlar, Germany). 

### 2.9. Pathogen (Fusarium) Leaf Detached and Wilt Assay

The fungal strain *Fol 1322* was obtained from the ICAR-Indian Agriculture Research Institute, New Delhi. The fungal culture was grown for 5 days at 25 °C in potato dextrose agar (PDA). For the pathogenic leaf disc assay, 10–15 large expanded leaves of 1-month-old tomato cv. AV were detached. Each leaf was soaked in sterile water-dipped Whatman filter paper to maintain the 70% RH. A 3-mm diameter agar plug containing mycelium of *Fol 1322* was put on the adaxial side of each Agrobacterium transformed and controlled wild-type (WT) (non-transformed) leaf segment [34,41]. The leaf segments covered with petri dishes were incubated at 25 °C under a 12 h:12 h light-dark photoperiod and then examined after 3–5 days of the post-infection day (PID). The symptoms of lesion development, necrosis, and wilting were recorded. The size of lesions (mm^2^) was measured using Image J software [42] by taking the average mean of three or five independent leaf discs. Similarly, broadly expanded leaves of stable transformed CRELs of *XSP10* and *SlSAMT* pathogen leaf disc assay were studied. Each experiment was repeated three times.

Using the root dip method [43], 1-month-old seedlings of WT and CRELs at GE_1_ generation were inoculated with virulent *Fol 1322*. The multiple disease resistant cv. Arka Abhed (AA) was used as a control check and mock (water only). For bioassay, CRELs tomato seedlings were placed in spore suspension (0.5 × 10^7^ spores/mL), and inoculated plantlets were immediately re-potted in the soil. A disease progression assessment was performed after three weeks of post-infection. Plant fresh weight (FW/g) and disease index (DI) scores were examined for 4 plants/treatment. The severity of the disease was determined using a DI grading system (0, no symptoms; 1, slightly sore or bent hypocotyl; 2, one or two brown vascular bundles in hypocotyl; 3, at least two brown vascular bundles and growth distortion; 4, all vascular bundles are brown, plant either dead or very small and wilted) [44]. 

### 2.10. Microscopy and Fungal Outgrowth Assay

For the microscopy assay, roots of 10–12-day-old tomato seedlings were treated with *Fol 1322* following the protocol of [45]. Water was used as a mock and multi-disease-resistant cv. AA was used as a control check. The roots of seedlings were gently washed with sterile water to remove the media component attached to the tips of the root system. The roots of clean seedlings were cut with a sterile blade and placed in a petri dish. The petri dish was filled with 20 mL of water, to which *Fol* spores were added to adjust to a final concentration of 0.5 × 107 spores/mL. Eventually, roots were inspected microscopically at 12 h and 24 h a day after *Fol* inoculation [46]. 

After scoring the disease severity, tomato stems were collected and surface-sterilized by water [4]. Under aseptic circumstances, stem pieces were rinsed with 70% ethanol and washed three times with sterile water. To this end, stem sections were taken at the positions of the cotyledon (C), second node (2), and fourth node (4). To avoid bacterial growth, the stem sections were placed on PDA plates containing antibiotics at the rate of 200 mg/L streptomycin and 100 mg/L penicillin. After keeping the plates for 5 days in the dark at 25 °C, the plates were examined [47]. The following scores were used to quantify colonization: 0 indicates that there is no fungal outgrowth from the stem piece, 1 indicates that there is fungal outgrowth at the crown or cotyledon level, and 2 indicates that there is fungal outgrowth at both the crown and crown cotyledon level [4]. 

### 2.11. Histochemical Cell Death Staining and Quantification 

Lactophenol trypan blue (TB) was used to detect the dying cells [48]. Leaves of *Fol 1322* infected were taken for cell death staining. Then, 10 mL of 85% lactic acid, 10 mL of phenol, 10 mL of distilled water, and 10 mg of trypan blue were used for preparing a stock solution of trypan blue (HiMedia). Diluting the stock at 1:1 with 95% ethanol yielded a workable solution. The leaves were incubated in a working solution for 1 h, then boiled for 1 min, cooled, and stored at room temperature overnight [48]. To remove chlorophyll content, the leaves were dipped in 95% ethanol and boiled for 8 min [49] and infected lesions on the leaves were examined and photographed [48]. The cell death/mm^2^ were marked and quantified using Image J software [42]. Each experiment was repeated three times. 

### 2.12. Detection of Hydrogen Peroxide (H_2_O_2_)

For hydrogen peroxide (H_2_O_2_) detection, the 3, 3-diaminobenzidine (DAB) staining method was used as a substrate [50,51]. First, 1 mg/mL DAB (HiMedia) solution was prepared, and the pH was adjusted to 3.6 with 0.1 N HCl. The DAB solution was dissolved for 1 h at 37 °C with vigorous shaking. Control and infected leaf samples were incubated in 10 mL of DAB solution overnight at 37 °C. After that, the excess DAB solution was rinsed away with distilled water. After boiling for 3 h in a fixative solution comprising ethanol and acetic acid in a 3:1 ratio, the stained leaf samples were incubated for 1 h in lacto-glycerol solution (lactic acid/glycerol/water, 1:1:1). The leaf samples were kept in microscopic slides and photographed [52]. The presence of H_2_O_2_/mm^2^ from WT (control) and CRISPR-edited necrotic leaves was quantified by using Image J software [42]. Each experiment was repeated three times. 

### 2.13. Statistical Analysis

For the measurement of lesion size and weight, a pairwise Student’s *t*-test (* *p* < 0.05, ** *p* < 0.01, *** *p* < 0.001) was used. The non-parametric Kruskal–Wallis test was performed for root colonization and disease severity using PRISM 9.0 Graph Pad software (https://www.graphpad.com/scientific-software/prism/ accessed on 10 April 2020). 

## 3. Results

### 3.1. Pre-Screening through Transient Analysis of the XSP10 and SlSAMT Single and Dual-Gene CRISPR/Cas9 Editing 

**Figure 1:**
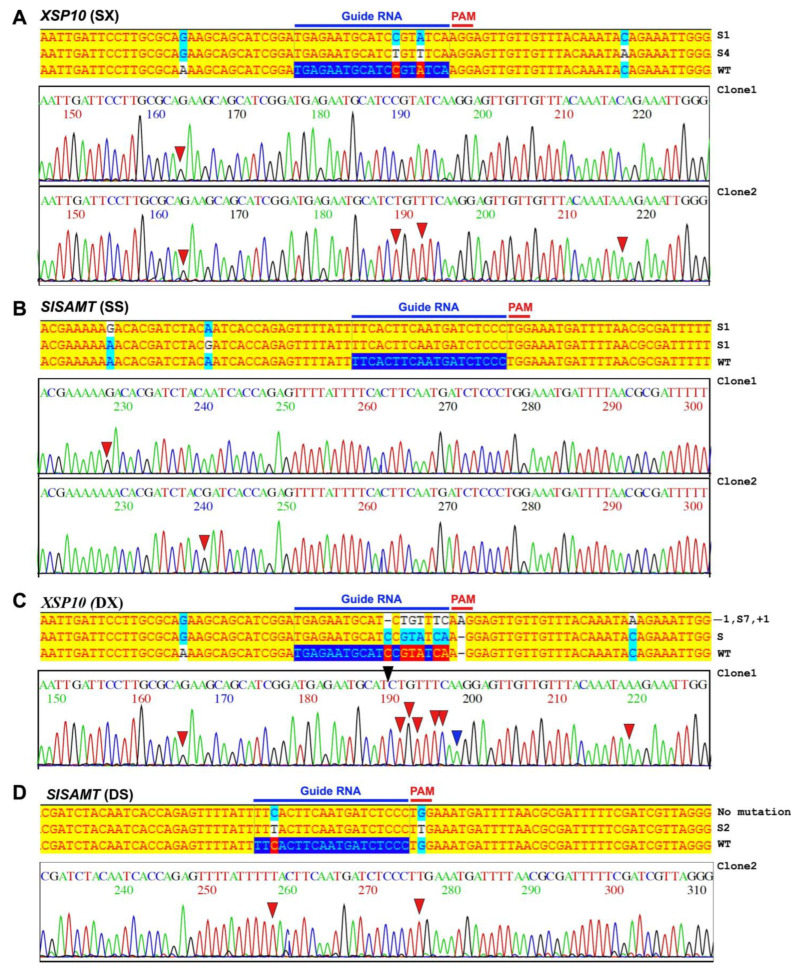
**Evaluation of single-gene and dual-gene CRISPR editing events of *XSP10* and *SlSAMT* through Sanger sequencing.** (**A**) *XSP10* single-gene editing (SX) events showing 1-bp (S1) and 4-bp (S4) substitution mutations in clone 1 and clone 2. Wild-type (WT) sequence of *XSP10* used as a reference sequence. The mutations in nucleotides of each clone indicated as ‘−’ for deletion, ‘+’ for insertion, and ‘s’ for substitution. The bold blue arrows underlined 19-nucleotide sgRNA seed sequences along with PAM sites in the reference gene sequence. In the chromatogram, the red arrow indicates ‘s’, blue indicate ‘+’, and black ‘−’ for insertion. (**B**) *SlSAMT* single-gene editing (SS) events showing 1-bp (S1) substitution mutation in clone 1 and 2. (**C**) Dual-gene editing (DXS) of *XSP10* (DX) showing 7-bp substitution (S7), 1-bp insertion (+1), and 1 bp-deletion (−1) observed in clone 1. (**D**) *SlSAMT* (DS) showing 2-bp substitution (S2) in clone 2 and no mutation in clone 1.

In order to identify an appropriate and effective guide RNA for stable line transformation, a transient experiment in the tomato protoplast system for single-gene (SX and SS) and dual-gene (DXS) CRISPR/Cas9 constructs of *XSP10* and *SlSAMT* genes was initially attempted. The schematic overview of sgRNA designing, CRISPR/Cas9 construct generation, and molecular confirmation in tomato leaf protoplasts is provided in Supplementary Figure S1. The presence of sgRNA sequences of *XSP10* and *SlSAMT* in the binary vector was confirmed by Sanger sequencing (Supplementary Figure S2A,B). Cas9 expression in tomato protoplasts was validated by PCR for single and dual-gene (DXS)-edited constructs (Supplementary Figure S2C), followed by sub-cloning, restriction digestion (Supplementary Figure S2D) and Sanger sequencing (Figure 1). The single-gene editing events for *XSP10* (SX) and *SlSAMT* (SS) were identified as follows: for SX, a base substitution mutation was found in clones 1 and 2 upstream and downstream of the target guide RNA region. (Figure 1A). Analogously, for SS, both clones 1 and 2 showed substitution mutations upstream of the PAM and target sgRNA region (Figure 1B). Other than substitution, no additional mutations were found in single-gene editing events. The following dual-gene editing (DXS) instances for *XSP10* (DX) and *SlSAMT* (DS) were evidenced: DX clone 1 exhibited substantial editing events, such as substitution, deletion, and insertion mutations. Clone 2 of DX showed a substitution mutation upstream of the target sgRNA (Figure 1C). However, only DS clone 2 exhibited substitution mutations at the PAM site (G was substituted by T) (Figure 1D). 

**Figure 2:**
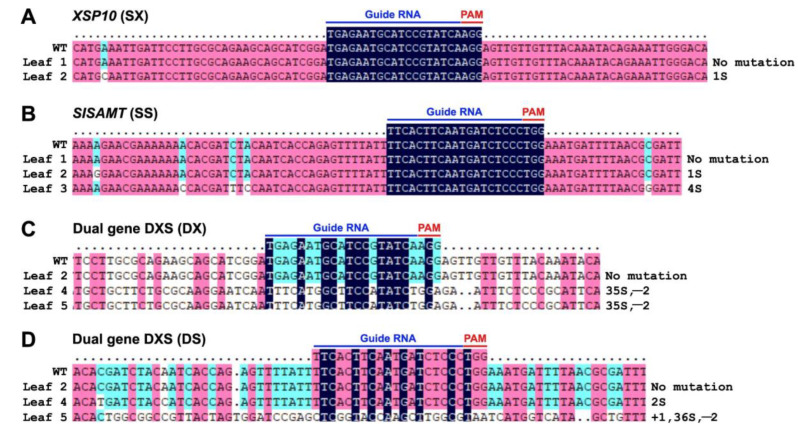
**Sanger sequencing confirmation of single and dual-gene DXS CRISPR editing events of *Fol 1322* tolerant leaves of tomato.** Insertion (+), deletion (−), substitution (s). (**A**) Leaf 2 of *XSP10*, Leaf 2 and 3 of *SlSAMT*. (**B**) Leaf 4, 5 of dual-gene editing of *XSP10* and *SlSAMT*. (**C**,**D**) SX: Single-gene editing of *XSP10*, SS: Single-gene editing of *SlSAMT*, DXS: Dual-gene editing of *XSP10* (DX) and *SlSAMT* (DS).

In the transient leaf disc assay, the positive transformants of single and dual-gene (DXS) constructs were confirmed through PCR analysis in leaves (Supplementary Figure S3A,B) as well as GUS expression analysis in fruits (Supplementary Figure S3C) and leaves (Supplementary Figure S3D,E). As shown in Figure 2, Sanger sequencing data indicated a multiplicity of editing events with INDEL and substitution mutations in the leaf disc. Leaf 2 of SX and SS single-gene editing showed a 1-bp substitution upstream of the guide RNA (Figure 2A). In leaf 3, 4-bp substitution mutations were observed upstream and downstream of the target gene of SS (Figure 2B and Figure S4). In dual-gene CRISPR editing (DXS), leaves 4 and 5 showed 2-bp deletion for DX, followed by 35-bp substitution (Figure 2C). In leaf 5, DS showed a 1-bp insertion, a 2-bp deletion, and a 36-bp substitution, while leaf 4 showed a 2-bp substitution (Figure 2D).

**Figure 3:**
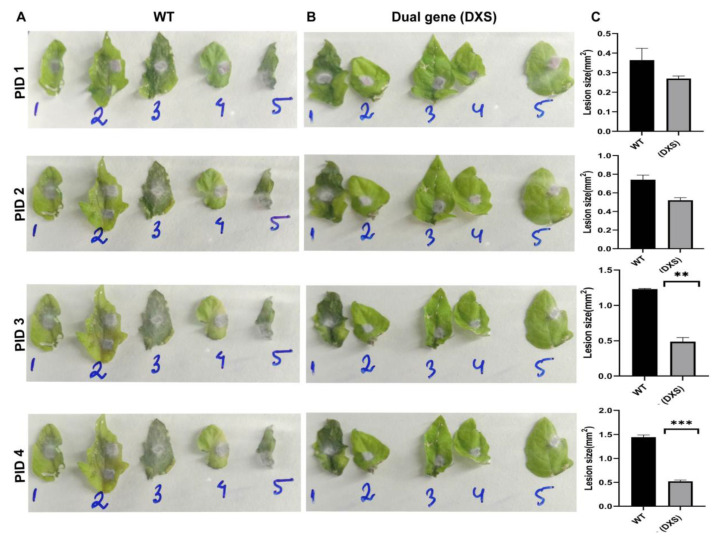
**Pathogen leaf disc assay for dual-gene CRISPR-edited tomato leaves infected with *Fol 1322* strain.** (**A**,**B**) WT and dual-gene (DXS) CRISPR/Cas9 positive transformants infected with *Fol 1322* strain. (**C**) Graphical representation of the average mean area of infected lesions of control and DXS in transient agro-infiltrated leaves. The area of lesions (mm^2^) was measured by Image J software and the average size of the lesion was taken for statistical significance. Bars represent the average means ± SE of measurements from 5 lesion spots from 5 different leaf discs of control (WT) and transformed dual-gene (DXS) CRISPR/Cas9 constructs. The asterisk denotes a significant difference determined by *t*-test (* *p* < 0.05, ** *p* < 0.01, *** *p* < 0.001). PID: post-infection day. WT: wild-type.

In response to *Fol 1322*, single-gene (SX and SS) and dual-gene (DXS)-edited leaves of tomato cv. AV developed necrotic lesions. Nevertheless, no significant variations in necrotic lesion progression were seen in single-gene edited *XSP10* and *SlSAMT* (variants) compared to WT (Supplementary Figure S5, Supplementary Table S3). Intriguingly, post-infection days (PID) 3 and 4 revealed disease symptoms including necrosis, yellowing, and curled WT leaf discs, in contrast to the dual-gene (DXS) construct (Figure 3A,B). On PID 3 and PID 4, the lesion size (mm^2^) of the dual-gene DXS construct was restricted by 75–80% as compared to WT (Figure 3C, Supplementary Table S4). Overall, the dual-gene CRISPR editing of DXS demonstrated significant tolerance response against *Fol 1322* infection compared to single-gene editing in the pathogen leaf disc assay (Figure 3). 

### 3.2. Generation of CRISPR-Edited Lines of GE_0_ and GE_1_ in S. lycopersicum cv. AV 

**Figure 4:**
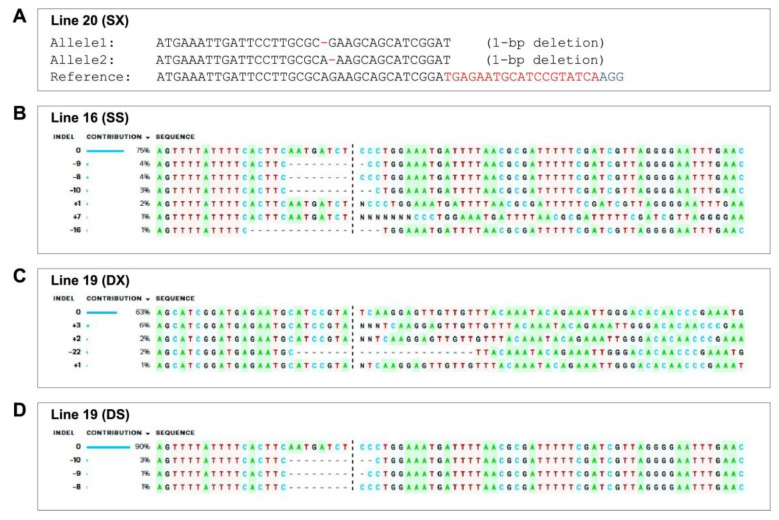
**Single and dual-gene DXS editing confirmation in tomato cv. AV at GE_0_ generation.** (**A**) Single-gene editing of SX-line 20 and SS-line 16 (**B**). (**C**) Dual-gene (DXS) line 19 editing of *XSP10* (DX) and *SlSAMT* (DS) (**D**); dashes represent deletions and N represents insertion at the guide RNA region. DS Decode M and ICE-Synthego software were used for decoding the PCR products. (+0) represents the WT. The sequencing chromatogram peaks of complicated chimeric plants with more than 2 editing events were unable to decode with DSDecode M software and were particularly analyzed with ICE-Synthego software.

Molecular analysis was performed to ascertain the editing events of single and dual-gene *XSP10* and *SlSAMT* in tomato cv. AV. Cas9 positivity was detected in 41 of 73 acclimatized GE_0_ plants (56.16%) (Supplementary Figure S6). Table 1 summarizes the efficiency of transformation and editing in GE_0_ plants. As per the sequencing results of the gene specific PCR products, nine plants were CRISPR-edited with a 34.61% editing efficiency. Additionally, 26 of the 41 Cas9 positive GE_0_ plants could be decoded using either DSDecodeM or ICE-Synthego software. Nine of the 26 plants showed CRISPR editing with insertions (N/+), deletions (−/dots), and substitutions (s) mutations. Three (SX) and five (SS) plants were identified for single gene editing, while only one (DXS) plant out of nine was detected for dual gene editing. SX, SS, and DXS showed editing efficiencies of 11.53%, 19.23%, and 3.8%, respectively. SX-line 20 had a 1-bp deletion in both alleles (bi-allelic) in a distinct region upstream of the sgRNA (Figure 4A and Figure S7). SX-line 40, on the other hand, showed a 3-bp insertion in one of the alleles (heterozygous) at the Cas9 cleavage sites. SX-line 34 exhibited INDELs in eight alleles (chimera) at the target sgRNA region. Likewise, SS-line 16 showed several INDEL mutations in the target sgRNA region, culminating in frame-shift of the open reading frame (ORF) and premature termination (Figure 4B). As a result, the lines of SS were concluded to be chimeras (Figure S8). DXS-line 19 showed chimeric mutations for the dual-gene-edited line (Figure 4C,D). Additionally, deletions were identified in the DXS-line 19 at the PAM site and the seed sequence of DX and DS, respectively. 

CRISPR-edited plants (GE_0_) were examined in the GE_1_ generation to investigate the transmission pattern of CRISPR/Cas9-mediated mutations in the *XSP10* and *SlSAMT* genes in tomato cv. AV. In the GE_1_ generation, the segregation analysis of three GE_0_ lines, viz., lines 20, 16, and 19, was performed (Table 2). PCR analysis revealed that all GE_1_ progenies carried T-DNA inserts in their genomes (Supplementary Figure S9). Among the selected 20 GE_1_ progenies, SX-20-12 and SX-20-13 were identified as bi-allelic. SX-20-5 and SX-20-14, on the other hand, showed 1-bp substitution at the target region and were confirmed as heterozygous. Among the 20 plants studied, genotypic analysis of SS-line16 progeny showed one heterozygous (SS-16-2), one bi-allelic (SS-16-7), and six chimeric plants (SS-16-8, SS-16-9, SS-16-10, SS-16-11, SS-16-14, SS-16-15). Only four of the dual-gene (DXS)-line 19 offspring (DXS-19-1, DXS-19-5, DXS-19-6, and DXS-19-10) were edited plants with chimeric and heterozygous genotypes (Supplementary Figure S10). DXS line-19-6, on the other hand, showed 1-bp substitution at the DX target sgRNA region and a frame-shift mutation in the target DS gene. 

### 3.3. Phenotypic Evaluation of Single and Dual-Gene Editing of XSP10 and SlSAMT upon Fol Infection in S. lycopersicum cv. AV

The single and dual-gene CRISPR-edited GE_1_ lines (SX: SX-20-5, SX-20-12, SX-20-13, SX-20-14; SS: SS-16-7, SS-16-8, SS-16-9, SS-16-10; DXS: DXS-19-1, DXS-19-9, DXS-19-6, DXS-19-10) were selected for phenotypic evaluation. Fol 1322 colonizes vigorously in the apex and epidermis of the root in WT compared to the CRELs of SX, SS, and DXS. SX and SS showed minimal root colonization by *Fol 1322* (*** *p* < 0.001) than WT (Supplementary Figure S11), although DXS had substantially less root colonization than SS and SX. In comparison to the control check AA, fungal hyphae colonization in root hairs was observed to be reduced in DXS-edited roots (Supplementary Table S5).

Mock-inoculated leaves in the leaf disc assay exhibited no symptoms of disease after 4 days. However, WT leaves showed more necrosis compared to single (SX and SS) and dual-gene (DXS) on the inoculated leaves after 4 days post infection. In SX and SS, there were a few dead cells (*** *p* < 0.001) on infected leaves. Nonetheless, as opposed to all other treatments (WT, SS, SX, AA), dual-gene (DXS) exhibited reduced death cells (Supplementary Figure S12A, Supplementary Table S6). The oxidative damage caused by *Fol 1322* infection in the leaves of CRELs of SX and SS plants produced less H_2_O_2_ than the WT (Supplementary Figure S12B, Supplementary Table S7). Interestingly, H_2_O_2_ production in dual-gene (DXS) was shown to be substantially lower than in WT, SX, SS, and AA.

**Figure 5:**
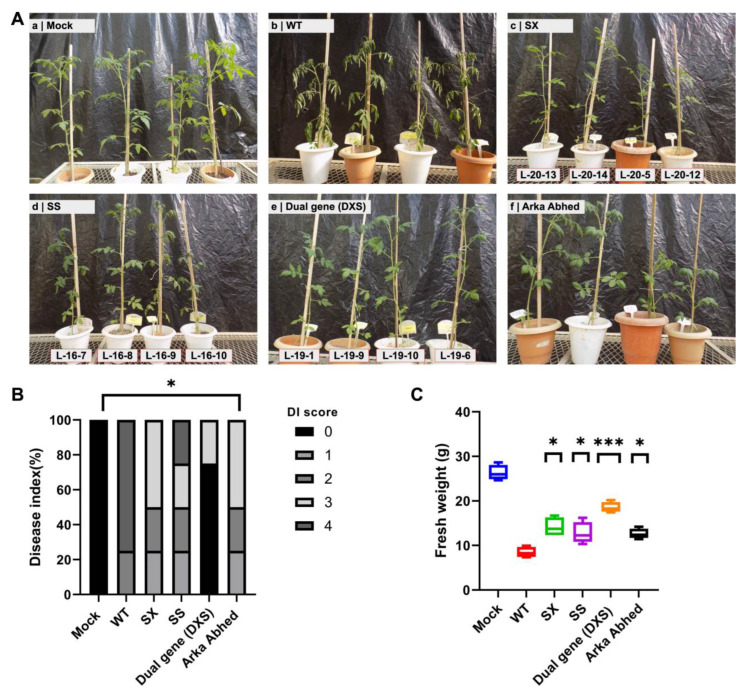
**Phenotypic evaluation of single and dual-gene CRISPR-edited lines showing tolerance response upon *Fol 1322* infection in 4-week-old tomato plants.** (**A**) Four-weeks old plants of WT and CRELS inoculated with water (mock) and *Fol 1322* after 21 days post-infection (PID). (**B**) Disease symptoms were scored by measuring the fresh weight above the cotyledon node and (**C**) disease index (0–4) of independent 4 plants/treatment. Plant fresh weight (FW) was subjected to a pairwise comparison Students *t*-test, whereas disease index was determined by non-parametric Kruskal–Wallis test (* *p* < 0.05, ** *p* < 0.01, *** *p* < 0.001).

The disease response in one month old plants after 21 days post-infection (DPI) was studied to check whether single and dual-gene CRELs of *XSP10* and *SlSAMT* alleviate Fusarium wilt disease susceptibility following *Fol 1322* infection. The studied lines exhibited increased fresh weight and decreased disease susceptibility in the following order: DXS > SX > SS > AA > WT (Figure 5A–C). The disease severity index (DI) score, which includes stunted growth, yellowing leaves, and wilting, was shown to be significantly lower in CRELs of single genes (SX and SS) compared to WT (Figure 5C, Supplementary Tables S8 and S9). Saliently, after 21 PID, dual-gene (DXS) lines showed significantly reduced wilting symptoms than all other treatments (WT, SX, SS, AA). Altogether, dual-gene (DXS)-edited lines demonstrated remarkably elevated Fusarium wilt disease tolerance. 

**Figure 6:**
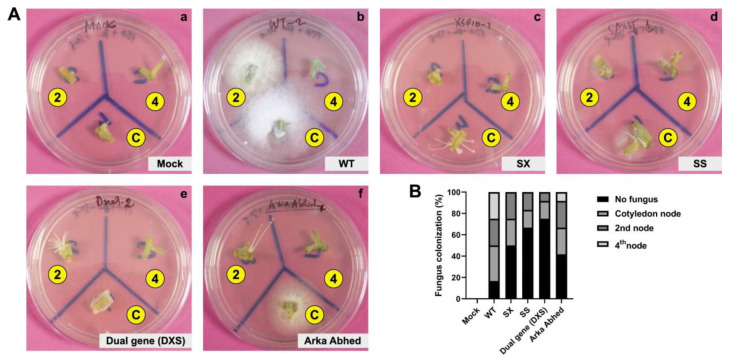
**An outgrowth of fungal pathogen *Fol 1322* in infected tomato stems**. (**A**) Representative picture of stem sections taken from cotyledon node (C) at **bottom**, 2nd node (2) at **top left** and 4th node (4) at **top right** of individual treated plants (n = 4) after incubation for 5 days on potato dextrose (PDA) plates with (penicillin + streptomycin) antibiotics. (**B**) Fungal colonization was expressed as a percentage of stem sliced infected of all stem pieces. All the data were statistically analyzed and no significant differences were observed (Student’s *t*-test * *p* < 0.05). L: line.

A fungal recovery experiment was performed to examine if disease susceptibility correlated with fungal colonization. The results showed that more than 70% of *Fol* colonized WT stems of the cotyledon node and second node, but single and dual-gene CRELs displayed reduced fungal proliferation. Nevertheless, no noticeable difference in *Fol* colonization was observed between single and dual-gene CRELs (Figure 6, Supplementary Table S10). The data imply that single and dual-gene CRELs of *XSP10* and *SlSAMT* induce genetic tolerance to *Fol 1322* of *S. lycopersicum* L., indicating a role as negative regulators of *Fol* tolerance. 

### 3.4. Analysis of Potential Off-Targets of the XSP10 and SlSAMT Genes of S. lycopersicum L.

The CCTop software was used to examine potential off-target (other than on-targeted sgRNA loci of *XSP10* and *SlSAMT*) effects of CRISPR/Cas9 editing in the entire tomato genome [27]. Off-target sites with less than 3–4 bp mismatches were considered for designing specific primers using vector NTI software (Life Technologies). The details of off-target efficacy, specificity, and mismatches are given in Supplementary Table S2. For the off-target investigation in GE_0_, CRISPR lines (SX-20, SX-34, DXS-19, SS-16, SS-23) were selected randomly. The off-target sites were PCR amplified using matched primer pairs and analyzed by Sanger sequencing Table 3, Supplementary Figure S13). No INDEL mutations were found at the off-target loci, suggesting that the CRISPR/Cas9 editing of *XSP10* and *SlSAMT* was target specific, and the *Fol* genetic tolerance response was mediated by the editing of *XSP10* and *SlSAMT* genes. 

## 4. Discussion

### 4.1. Validation of CRISPR/Cas9 Editing at the Transient Level Showed Fol 1322 Tolerance in S. lycopersicum L.

Stable transformation in any given plant species to study the CRISPR-induced mutations and its subsequent heritability to the next generation is time-consuming. A precise and pragmatic way of evaluating the efficacy of different sgRNAs-Cas9 complex for CRISPR-mediated mutagenesis is single-cell (protoplast) transformation. Since a particular gene is present in two copies in a diploid cell (or four copies in an amphidiploid cell like *N. tabacum*), mutated copies of the gene will make up 50% (for heterozygous cells) or 100% (for homozygous or bi-allelic cells) of the targeted gene [23]. In the current study, we thus started with protoplast transformation before stepping on to stable line generation. Dual gene editing (DXS) resulted in a range of mutation types in terms of insertion, deletion, and substitution as compared to single gene editing (SX and SS), which mostly exhibited base substitution. Parallel to our findings, Sun and colleagues reported single base substitution in protoplast using the CRISPR/Cas9 system targeting the *Glyma12g37050* gene of soybean [24]. Similarly, Li et al. (2013) reported multiple mutations that targeted the *AtPDS3* and *NbPDS* genes in the protoplasts of tobacco and Arabidopsis, respectively [53]. Protoplasts from at least five crop species (rice, wheat, maize, lettuce, and tomato), in addition to Arabidopsis and tobacco, have been used to evaluate gene editing reagents using CRISPR/Cas9-based systems [54,55,56,57].

Several studies have proven that the transient leaf disc assay is a comparably rapid technique of verifying the efficiency of sgRNA *in planta* before proceeding to generating stable genome-edited plants [58]. Transient gene expression by agro-infiltration has been a choice for functional studies in tomato fruits and leaves [33]. Previously, in stable transgenic tomato lines, the detached leaf bioassay infected with *Fol* was carried out [41]. In the present research, the lesion size in the leaf disc infected with *Fol 1322* was observed to be significantly larger in the wild-type (WT) than in the DXS, although the SX and SS exhibited subtle phenotypes. Following *Fol 1322* infection, DXS revealed a 30% drop in the development of necrotic lesions. The strong phenotypic response in DXS could be due to the occurrence of INDEL mutations at the target sgRNA region. 

### 4.2. CRISPR/Cas9 Induced Stable Gene Editing and Heritability in GE_1_ Generation

Our findings indicate that in the GE_0_ generation, the average transformation efficiency produced by gRNA:Cas9 was 56.16%, and the cumulative (SX, SS, and DXS) editing efficiency/mutation rate was 34.60%. It has been previously reported that when the CaMV 35S promoter is used to drive the expression of Cas9 endonuclease in dicotyledons, the mutation frequency ranges significantly from 26–95% [59,60,61]. 

Three lines from the GE_0_ generation (SX-line 20, SS-line 16, and DXS-line 19) were selected for inheritance evaluation of mutations in the GE_1_ generation. Two SX-20 GE_1_ lines exhibited bi-allelic mutations, while two others carried heterozygous mutations. Likewise, among 20 GE_1_ lines of SS-16, one heterozygous plant, one bi-allelic plant, and six chimeric plants were detected. In the instance of the dual gene (DXS)-19 line, four progenies inherited chimeric and heterozygous mutations. The frequency of mutations (editing efficiency), which we observed in GE_1_ lines, signifies the level of somatic mutations and does not provide a precise estimation of the occurrence of germ-line mutations at GE_0_ or GE_1_, which is indicative of the fact that germ-line cells make up a considerably small portion of a plant’s total cell population [59]. Thus, our study suggests that detailed screening of heritable mutations should be carried out in the T_2_ or later generations. 

Based on genotyping data, we concluded that chimeric mutations are prevalent in the GE_0_ generation of *S. lycopersicum* stable CRISPR-edited plants. Similar results have been observed in other plants, including *Arabidopsis* [61], tomato [62], rice [63], and barley [64], which are consistent with our findings. Low editing efficiency in GE_0_ somatic cells, which could not be transmitted by all reproductive tissues producing seeds for the subsequent generation, might have been the major cause of such an occurrence [59,65]. The increased editing frequency with several mutant alleles observed in GE_1_ plants evidenced that the SpCas9-sgRNA complex was continuously expressed in GE_0_ plants throughout the developmental phases [66]. 

The CRISPR/Cas9 system has been reported to induce a few off-target effects in model plants like Arabidopsis [59], rice [67], and tomato [68]. Generally, off-targets with less than 2–3 bp mismatch at the seed sequence have fewer chances for the sgRNA/Cas9 complex to recognize the on-target sites [69]. No off-target alterations were noted in this study, demonstrating the phenotypic effect is merely the result of editing targeted genes and pointing to the high specificity of the CRISPR/Cas9 system in tomato plants. The targeting specificity of Cas9/sgRNA is determined by several parameters. The most crucial factor for defining the binding specificity of the sgRNA guiding sequence is the PAM-proximal region [70]. Therefore, the most efficient strategy to reduce off-target occurrences is to design a very specific target sequence. For model and crop plants, several bioinformatic methods have been created that can give highly precise sgRNAs [71]. 

### 4.3. CRISPR-Edited Lines (CRELs) of XSP10 and SlSAMT Reduce the Susceptibility of Tomato to Fusarium Wilt

Insights from earlier investigations have led to the proposal that *SlSAMT* and *XSP10* are two significant negative regulatory candidate genes, the expression of which makes the tomato plant susceptible to Fusarium wilt. The fungal pathogen (*Fol*) primarily enters into the plant system through roots [72] and colonizes the xylem tissues [73]. The primary effect of root colonization by *Fol* is to impede the flow of water via the xylem vessels, which causes the plant to exhibit symptoms of wilting [3,73]. 

In the current study, CRELs of SX, SS, and DXS exhibited much lower fungal colonization in the root epidermis and cortex (Supplementary Figure S11) than the WT. Consequently, at the phenotypic level, there were no apparent symptoms of wilting, and the development of necrotic lesions was relatively lower in the SX, SS, and DXS plants than in WT plants (Figure 5). This might be owing to the loss-of-function of the *XSP10* and *SlSAMT* genes, which prevent fungal hyphae from penetrating the root epidermis and causing wilting symptoms [26]. The fungus’s diminished ability to colonize the root surface of CRELs might be due to two possibilities. One possibility is that the fungus requires XSP10 as a compatibility target in order to fully develop the disease. In the apparent lack of this target, the fungus’ capacity to colonize the plant and cause disease is restricted. In the same way as elicitins secreted by pathogenic *Phytophthora* or *Pythium* species have been proposed as sterol transporters, *XSP10* may be implicated in the transport of essential lipid molecules from plant membranes to the pathogen [10]. In the second possibility, *XSP10* may be a part of a signaling cascade that activates host defense mechanisms following pathogen recognition. The tomato never ripe (NR) mutant, which does not exhibit symptoms while being equally colonized by the disease as WT plants, is a clear indication of how the plant controls the process of symptom development [74]. If *XSP10*, as a positive regulator, is implicated in a systemic signaling critical for symptom development, then inactivating it will result in reduced symptom development rather than heightened disease resistance. If, on the other hand, XSP10 encodes a negative regulator, then inhibiting *XSP10* is expected to enhance host resistance, limiting *Fol* colonization and symptom progression [9]. Recently, the vascular colonization of tomato plants harboring three distinct categories of resistance (R) gene types by *Fol* was studied. Vascular colonization was noticed in all cases, despite the fact that the immune receptors (I and I-3) that are located on the plasma membrane impeded colonization more significantly than the intracellular receptor (I-2) [45]. Analogously, de Lamo et al. (2018) reported that the proportion of fungal proliferation in a resistant plant is restrained, and fungal proteins in diseased plants’ xylem sap cannot be quantified [4]. These results are in accordance with the low count of hyphae that appear in xylem vessels of a resistant tomato cultivar [43]. Additionally, Pu and co-workers documented that there was negligible vascular colonization after inoculating *Fusarium oxysporum* f.sp. *conglutinans* into resistant cabbage roots, and no fungal proteins were found in the xylem sap [75].

Systemic acquired resistance (SAR) is caused by the buildup of SA in both local and systemic tissues, which is brought on by the activation of both the local and systemic host defense mechanisms [76]. By converting SA to MeSA, or in other words, by impairing SA signaling, *SlSAMT* modulates SA homeostasis [13]. As a result, it weakens the host’s defensive mechanism against *Fol* infection [13]. Based on the findings of this work, we predicted that CRISPR editing of *SlSAMT* in conjunction with *XSP10* could result in decreased *Fol* colonization in root tissue. In parallel to our results, Ament and colleagues reported that the RNAi-mediated silencing of *SAMT* in tomato significantly reduced the susceptibility of the plant to virulent strains of *F. oxysporum* f. sp. *lycopersici* [13]. *A. thaliana* genotypes deficient in SA signaling were shown to be more vulnerable to *F. oxysporum* [77], while *F. oxysporum* f. sp. *lycopersici* infection of tomato is primarily reliant on SA levels in the host, with elevated SA levels correlating to fewer disease symptoms [78]. Overall, the functional analysis of dual and single gene editing of *XSP10* and *SlSAMT* exhibited tolerance to *Fol* compared to the WT, indicating that XSP10 and SlSAMT interact to impair the tolerance of tomato to the Fusarium wilt pathogen.

The HR is generally associated with a localized burst of reactive oxygen species (ROS) and programmed plant cell death (PCD) when pathogen effectors are recognized by specialized host immune receptors [79]. Under biotic stress, ROS production generally induces lipid peroxidation, which leads to oxidative damage to the cell membrane [80]. We assayed the generation of ROS upon *Fol 1322* infection on CRELs and WT plants of *S. lycopersicum* by using DAB staining procedure. Additionally, we examined the cell death in the WT and CRELs using trypan blue reagent. In our observation, dual-gene (DXS) editing lines of *XSP10* and *SlSAMT* in tomato cv. AV exhibited fewer cell deaths and lower ROS accumulation on the surface of the leaves compared to WT (Supplementary Figure S12). In order to detoxify ROS accumulation, different antioxidant enzymes, such as superoxide dismutase (SOD), catalase (CAT), and ascorbate peroxidase (APx), have been reported to be involved in ROS metabolism during *Fol* infection [79]. CAT can effectively remove most of the H_2_O_2_, while APX can scavenge H_2_O_2_, which is inaccessible for CAT because of the high affinity towards H_2_O_2_ [80]. Exogenous SA application in *Fol*-infected tomato plants has been shown to reduce H_2_O_2_ accumulation and lipid peroxidation during Fusarium wilt disease [79]. Indeed, PR-1 and other defense-related proteins often accumulate at the sites of *Fol*-infected tomato plants [44]. Thus, ROS activity increases at Fusarium infection sites, and fungal colonization may activate host transcriptional factors, defense-related genes, and antioxidant enzymes [81]. In this context, it is noteworthy that loss-of-function of a dual-gene (DXS) may augment a defense response in CRISPR plants. During the *Fol*-Arabidopsis interaction, it was reported that cell death mediated by ROS together with an impaired SA signaling pathway resulted in disease development [80].

In order to check the fungal progression in the stem, we performed a root dip assay followed by fungal outgrowth/recovery assay. After 21 PID, CRELs of the *XSP10* and *SlSAMT* genes were found to be resilient against the Fusarium wilt pathogen, with no obvious disease symptoms. The DXS lines were found to be more tolerant to Fusarium wilt pathogen than the SX and SS lines. WT plants, on the other hand, had significant wilting symptoms in the root dip assay. Aside from that, the CRISPR/Cas9-edited lines exhibited a higher FW than the WT plants. FW was substantially higher in SX and SS lines than in WT plants. However, compared to the SX and SS lines, the FW of DXS lines was significantly higher. This clearly indicates the loss-of-function of the two negative regulatory genes that resulted in decreased fungal colonization in the CRISPR/Cas9-mediated single and dual-gene-edited plants [9].

Analogously, in the fungal outgrowth/recovery assay, the CRELs of *XSP10* and *SlSAMT* genes showed reduced fungal colonization as compared to the WT plants, indicating the negative regulatory role of these two genes in tomato against the Fusarium wilt pathogen. These results are consistent with the findings of Krasikov et al. (2011) [9] and Ament et al. (2010) [13], where they have reported diminished *Fol 1322* infection in tomato due to silencing of *XSP10* and *SlSAMT*. 

## 5. Conclusions and Future Prospects

In comparison to single-gene editing, stable dual-gene CRISPR/Cas9 editing of *XSP10* and *SlSAMT* in disease-susceptible tomato cv. AV demonstrated substantial resistance to Fusarium wilt. An extensive molecular genetic analysis of transient and stable lines (GE_1_) demonstrated that *XSP10* and *SlSAMT* are exerting a negative regulatory role in conferring genetic tolerance to the tomato Fusarium wilt disease. While molecular analysis and stress tolerance analyses of the potential genome-edited lines were undertaken in the GE_0_ and GE_1_ generations, we have not yet obtained any homozygous-edited lines, which assert that our prospective extended experimentation would be primarily focused on extensive molecular screening of the genome-edited mutant plants in successive generations, unless we obtain a homozygous-edited line. Furthermore, the variations in mutation types observed might well be ascribed to multiple T-DNA integration in GE_0_ plants. The establishment of transgene-free genome-edited tomato lines capable of conferring resistance to the disease fusarium wilt would thus be critical. Furthermore, on March 30, 2022, the Ministry of Environment, Forest, and Climate Change released an Office Memorandum (OM No. F. No. C-12013/3/2020-CS-III, dated 30 March 2022) exempting SDN-1 and SDN-2 categories of Genome-Edited Plants that are devoid of foreign inserted DNA from the provisions of Rules 7 to 11 (both inclusive) of the Rules 1989 of the EPA, 1986. Hence, the findings of the current study would be significant in extending the research toward experimental field evaluation for heritability and stability of Fusarium wilt disease tolerance of CRISPR-edited tomato lines (GE_2_ and GE_3_), licensing and commercial prospects. The study also lays a solid foundation for analyzing similar negatively regulated genes for biotic or abiotic stress tolerance in other economically important crop plants. 

## Figures and Tables

**Table 1:** Summary of Cas9 positive and editing efficiency in GE_0_ lines of *S. lycopersicum* cv. AV.

No. of Plants Examined	Cas9 Positive Efficiency (%)	Editing Efficiency (%)			
		Single Gene (SX)	Single Gene (SS)	Dual Gene (DXS)	Combined (SX, SS and DXS)
73	56.16% (41/73)	11.53% (3/26 *)	19.23% (5/26 *)	3.8% (1/26 *)	34.6% (9/26 *)

* 15 plants were not decoded with either of the DS Decode M or ICE- Synthego software.

**Table 2:** Segregation of CRISPR/Cas9-edited lines in GE_1_ progeny.

	GE_0_ Segregation			GE_1_ Segregation	
Target Gene	Lines	Cas9	Mutation	Mutation	Cas9/T-DNA
*XSP10* (SX)	20	+	Bi-allelic	2 Bi/2 He	+
*XSP10* (SX)	34	+	Chimera	NA	NA
*XSP10* (SX)	40	+	Heterozygote	NA	NA
*XSP10* (DX)	19	+	Chimera	3 Chi/2 He	+
*SlSAMT* (SS)	16	+	Chimera	1 Bi/1 He/6 Chi	+
*SlSAMT* (SS)	7	+	Chimera	NA	NA
*SlSAMT* (SS)	23	+	Chimera	NA	NA
*SlSAMT* (SS)	33	+	Chimera	NA	NA
*SlSAMT* (SS)	52	+	Chimera	NA	NA
*SlSAMT* (DS)	19	+	Chimera	3 Chi/2 He	+

(**Note:** ‘He’ stands for heterozygotes, ‘Chi’ for chimera, ‘Bi’ for Bi-allelic, and ‘WT’ for wild-type genotypes of segregating plants; (+) and (−) represents Cas9/T-DNA present and absent in the progenies respectively; NA not analyzed in GE_1_ generation).

**Table 3:** Sanger sequencing results of potential off-targets of *XSP10* and *SlSAMT* in GE_0_ CRELs.

Target	Potential Off-Target Sequence	No. of Mismatch Bases	Total No. of Plants Tested	No. of Mutation
*Solyc05g016300*	TTTGAATGCTTCCATATCAGGG	4	5	0
*Solyc12g016150*	TGATATTGGACCCGTATCACGG	4	5	0
*Solyc02g032110*	TTATATTGAATCCGTATCA CGG	4	5	0
*Solyc01g057280*	ATCACTAAAATGTTCTCCCAGG	4	5	0
*Solyc09g060120*	CTCATTTGAATGATCACCCAGG	4	5	0

## Data Availability

Not applicable.

## Supplementary Materials

The following supporting information can be downloaded at: https://www.mdpi.com/article/10.3390/genes14020488/s1, Figure S1. Overview of sgRNA design, CRISPR cloning into expression vector, protoplast transformation, and sequencing for editing events. Figure S2. Molecular confirmation of CRISPR transformants in tomato protoplasts. Figure S3. Molecular and histochemical confirmation of positive transformants of single and DXS. A–D. Figure S4. Chromatograms confirming the CRISPR editing events of single and dual-gene in tomato leaves tolerant to *Fol 1332*. Figure S5. Pathogen leaf disc assay for single-gene CRISPR-edited tomato leaves infected with *Fol 1322* strain. Figure S6. Positive plant transformants of CRISPR/Cas9 construct. Figure S7. Chromatograms confirming the CRISPR editing events of single and dual-gene of *S. lycopersicum* cv. Arka Vikas at GE_0_ generation. Figure S8. Sanger sequencing and chromatograms confirming the CRISPR editing events of SX and SS of GE_0_ generation from *S. lycopersicum* L. Figure S9. Presence of T-DNA integration in GE_1_ progeny. Figure S10. Sanger sequencing and chromatograms confirming the CRISPR editing events of single (SX and SS) and dual-gene (DXS) of GE_1_ generation from *S. lycopersicum* L. Figure S11. *Fol 1322* colonization in 10–12-day-old seedlings of tomato cv. AV at 12 h and 24 h post-inoculation. Figure S12. Single and dual-gene CRELS of *XSP10* and *SlSAMT* showed reduced necrotic lesions upon *Fol 1322* infection in tomato leaves. Figure S13. Potential off-target sites generated in GE_0_ CRELS by Sanger sequencing method. Table S1. Primers used in this study. Table S2. Summary of sgRNA design of target genes *XSP10* and *SlSAMT* along with off-target details. Table S3. Data measurement of lesion size upon *Fol 1322* infection in control and single-gene constructs using Image J software. Table S4. Data measurement of lesion size upon *Fol 1322* infection in control and dual-gene (DXS) constructs using Image J software. Table S5. Statistical data analysis of root colonization in 10–12-day-old seedlings of tomato cultivar Arka Vikas using Image J software. Table S6. Data measurement of cell death/mm^2^ in infected tomato leaves upon *Fol 1322.* infection in WT, single (SX and SS) and dual-gene (DXS) constructs using Image J software. Table S7. Data measurement of H_2_O_2_ release/mm^2^ in infected tomato leaves upon *Fol 1322.* infection in WT, single (SX and SS), and dual-gene (DXS) constructs using Image J software. Table S8. Data measurement of fresh weight (g) of Mock, WT, single and dual-gene (DXS), and Arka Abhed upon *Fol 1322*-infected tomato plants. Table S9. Data measurement of disease severity of single and dual-gene (DXS) in 4-week-old tomato infected plants. Table S10. Data measurement of fungus colonization in WT and CRELS upon *Fol 1322* infection in tomato (*S. lycopersicum* L.).

## Abbreviations

SAR: systemic acquired resistance; XSP: xylem sap protein; SAMT: salicylic acid methyl transferase; MeSA: methyl salicylate; CRISPR/Cas9: clustered regulatory interspaced short palindromic repeats/CRISPR associated protein 9; DSB: double-stranded break; NHEJ: non-homologous end joining; HDR: homology-directed repair; JA: jasmonic acid; SA: salicylic acid; CRELs: CRISPR-edited lines.
