# Supplementary material for: CRISPR/Cas9-Mediated Mutation in XSP10 and SlSAMT Genes Impart Genetic Tolerance to Fusarium Wilt Disease of Tomato (Solanum lycopersicum L.)
Source: Genes (Basel). 2023 Feb 14;14(2):488. doi: 10.3390/genes14020488 (PMC9956927; doi:10.3390/genes14020488)
Supplement: Supplementary file 1 [file genes-14-00488-s001.zip › genes-2122692-supplementary.pdf]

genes

MDPI

Supplementary Materials

CRISPR/Cas9-Mediated Mutation in *XSP10* and *SlSAMT* Genes Impart Genetic Tolerance to Fusarium Wilt Disease of Tomato (*Solanum lycopersicum* L.)

Supplementary Information

Supplementary Figures:

Supplementary Figure S1. Overview of sgRNA design, CRISPR cloning into expression vector, protoplast transformation and sequencing for editing events.
Supplementary Figure S2. Molecular confirmation of CRISPR transformants in tomato protoplasts.
Supplementary Figure S3. Molecular and histochemical confirmation of positive transformants of single and DXS. A-D.
Supplementary Figure S4. Chromatograms confirming the CRISPR editing events of single and dual-gene in tomato leaves tolerant to *Fol 1332*.
Supplementary Figure S5. Pathogen leaf disc assay for single-gene CRISPR edited tomato leaves infected with *Fol 1322* strain.
Supplementary Figure S6. Positive plant transformants of CRISPR/Cas9 construct.
Supplementary Figure S7. Chromatograms confirming the CRISPR editing events of single and dual-gene of *S. lycopersicum* cv. Arka Vikas at GE_0 generation.
Supplementary Figure S8. Sanger sequencing and chromatograms confirming the CRISPR editing events of SX and SS of GE_0 generation from *S. lycopersicum* L.
Supplementary Figure S9. Presence of T-DNA integration in GE_1 progeny.
Supplementary Figure S10. Sanger sequencing and chromatograms confirming the CRISPR editing events of single (SX and SS) and dual-gene (DXS) of GE_1 generation from *S. lycopersicum* L.
Supplementary Figure S11. *Fol 1322* colonization in 10-12 days old seedlings of tomato cv. AV at 12 h and 24 h post-inoculation.
Supplementary Figure S12. Single and dual-gene CRELS of *XSP10* and *SlSAMT* showed reduced necrotic lesions upon *Fol 1322* infection in tomato leaves.
Supplementary Figure S13. Potential off-target sites generated in GE_0 CRELS by Sanger sequencing method.

Supplementary Tables:

Supplementary Table S1. Primers used in this study.
Supplementary Table S2. Summary of sgRNA design of target genes *XSP10* and *SlSAMT* along with off-target details.
Supplementary Table S3. Data measurement of lesion size upon *Fol 1322* infection in control and single-gene constructs using Image J software.
Supplementary Table S4. Data measurement of lesion size upon *Fol 1322* infection in control and dual-gene (DXS) constructs using Image J software.

Supplementary Table S5. Statistical data analysis of root colonization in 10-12 days old seedlings of tomato cultivar Arka Vikas using Image J software.

Supplementary Table S6. Data measurement of cell death/mm^2 in infected tomato leaves upon *Fol 1322.* infection in WT, single (SX and SS) and dual-gene (DXS) constructs using Image J software.

Supplementary Table S7. Data measurement of H_2O_2 release/ mm^2 in infected tomato leaves upon *Fol 1322.* infection in WT, single (SX and SS) and dual-gene (DXS) constructs using Image J software.

Supplementary Table S8. Data measurement of fresh weight (g) of Mock, WT, single and dual-gene (DXS), and Arka Abhed upon *Fol 1322* infected tomato plants.

Supplementary Table S9. Data measurement of disease severity of single and dual-gene (DXS) in 4 weeks old tomato infected plants.

Supplementary Table S10. Data measurement of fungus colonization in WT and CRELS upon *Fol 1322* infection in tomato (*S. lycopersicum* L.).

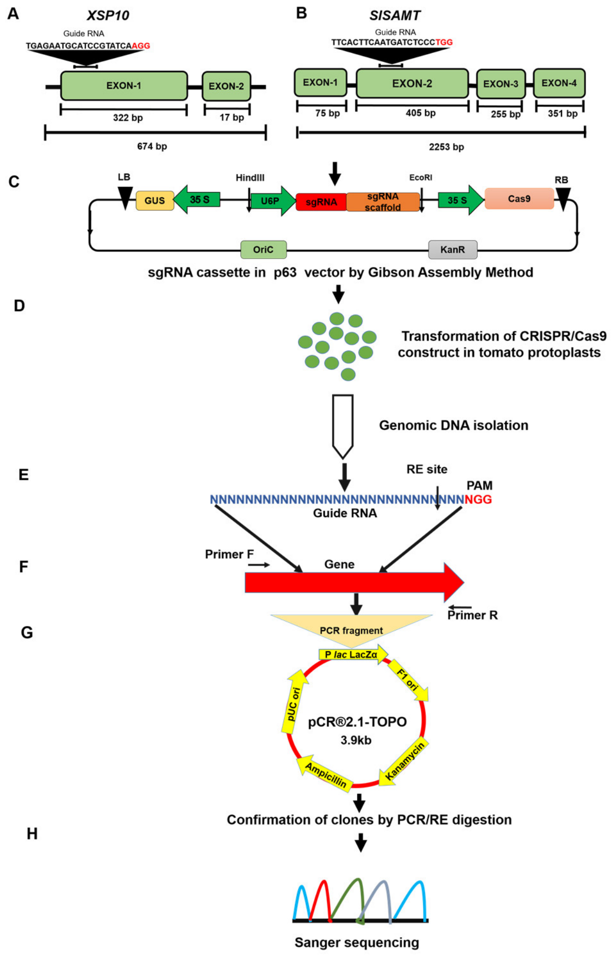

Supplementary Figure S1. Overview of sgRNA design, CRISPR cloning into the p63 expression vector, protoplast transformation and sequencing for editing events. sgRNA designing of *XSP10* **(A)** and *SlSAMT* (**B**).**C.** sgRNA cassettes assembled in p63 vector by Gibson assembly method. **D.** The single-gene and dual-gene CRISPR constructs transformed into tomato protoplast cells. **E.** PCR confirmation of expression of Cas9. **F.** PCR fragments ligated and cloned into pCR2.1 T/A cloning vector and confirmation by PCR and restriction digestion **(G). H.** Detection of CRISPR editing events through Sanger sequencing.

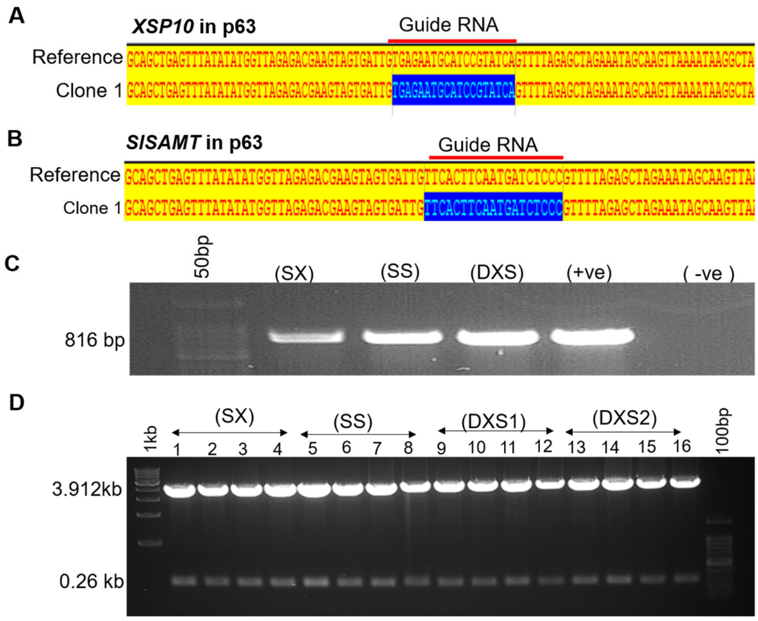

Supplementary Figure S2. Molecular confirmation of CRISPR transformants in tomato protoplasts. Confirmation of sgRNA sequence of *XSP10* **(A)** and *SlSAMT* **(B)** in p63 binary expression vector through Sanger sequencing. **C.** Detection of Cas9 in transformed tomato protoplasts through PCR of single (SX and SS) and dual gene DXS **(D)** restriction digestion confirmation of positive clones of single and dual gene PCR product into sub-cloning vector pCR2.1 with enzyme EcoRI.

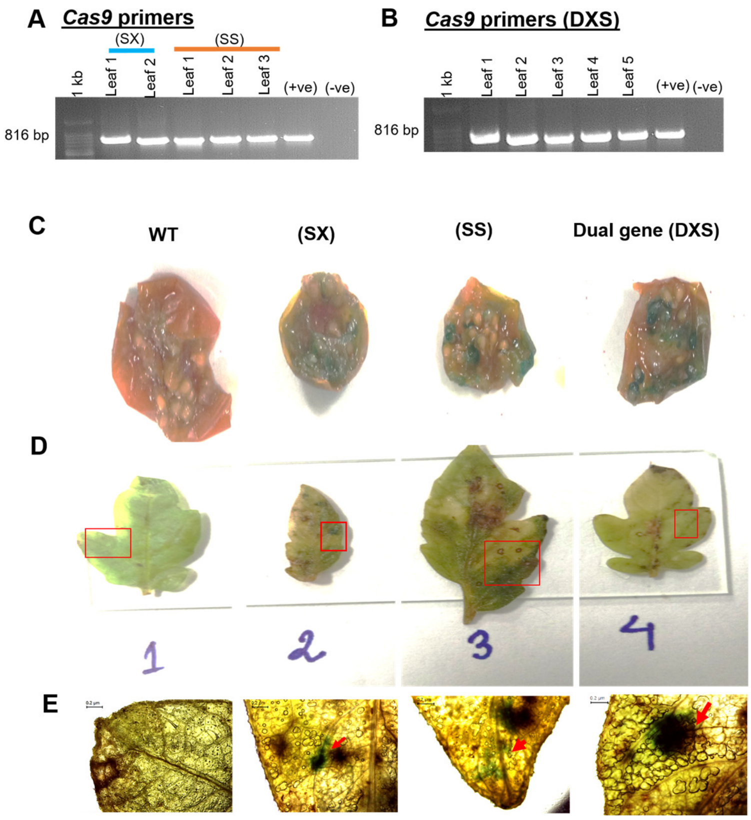

Supplementary Figure S3. Molecular and histochemical confirmation of positive transformants of single and DXS. A-B. PCR confirmation using Cas9. Histochemical GUS staining of single and dual-gene (DXS) CRISPR/Cas9 construct of *XSP10* and *SlSAMT* in fruit tissues (**C**) and leaf tissues (**D**) of tomato cultivar AV.

E. Expanded light microscopy images of leaves showing GUS staining for better clarity. No GUS activity was observed in control (water treated) fruits and leaves. SX: Single-gene editing of *XSP10*, SS: Single-gene editing of *SlSAMT*, DXS: Dual-gene editing of *XSP10* and *SlSAMT*.

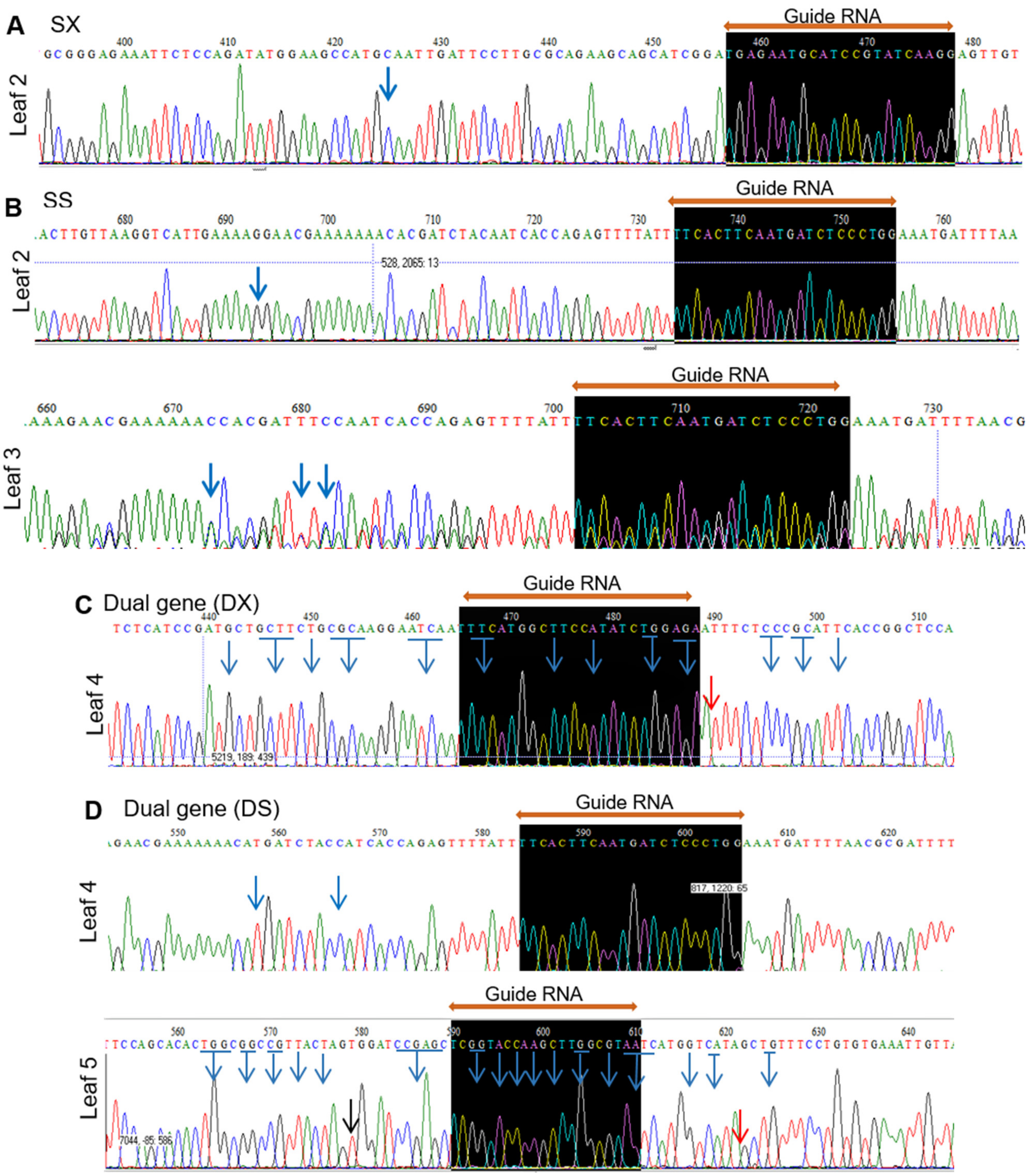

Supplementary Figure S4. Chromatograms confirming the CRISPR editing events of single and dual-gene in tomato leaves tolerant to *Fol 1332*. A. Leaf 2 of single gene *XSP10* **(SX)**, **B.** Leaf 2 , leaf 3 of single gene *SlSAMT* (SS), **C.** Leaf 4 of dual gene (DX) and **D**. Leaf 4, Leaf 5 of dual gene (DS). The blue arrow

indicates base substitution (s), black arrow indicate insertion (+) and red indicate deletion (-). SX: Single gene editing of *XSP10*, SS: Single gene editing of *SlSAMT*, DXS: Dual gene editing of *XSP10* (DX) and *SlSAMT* (DS).

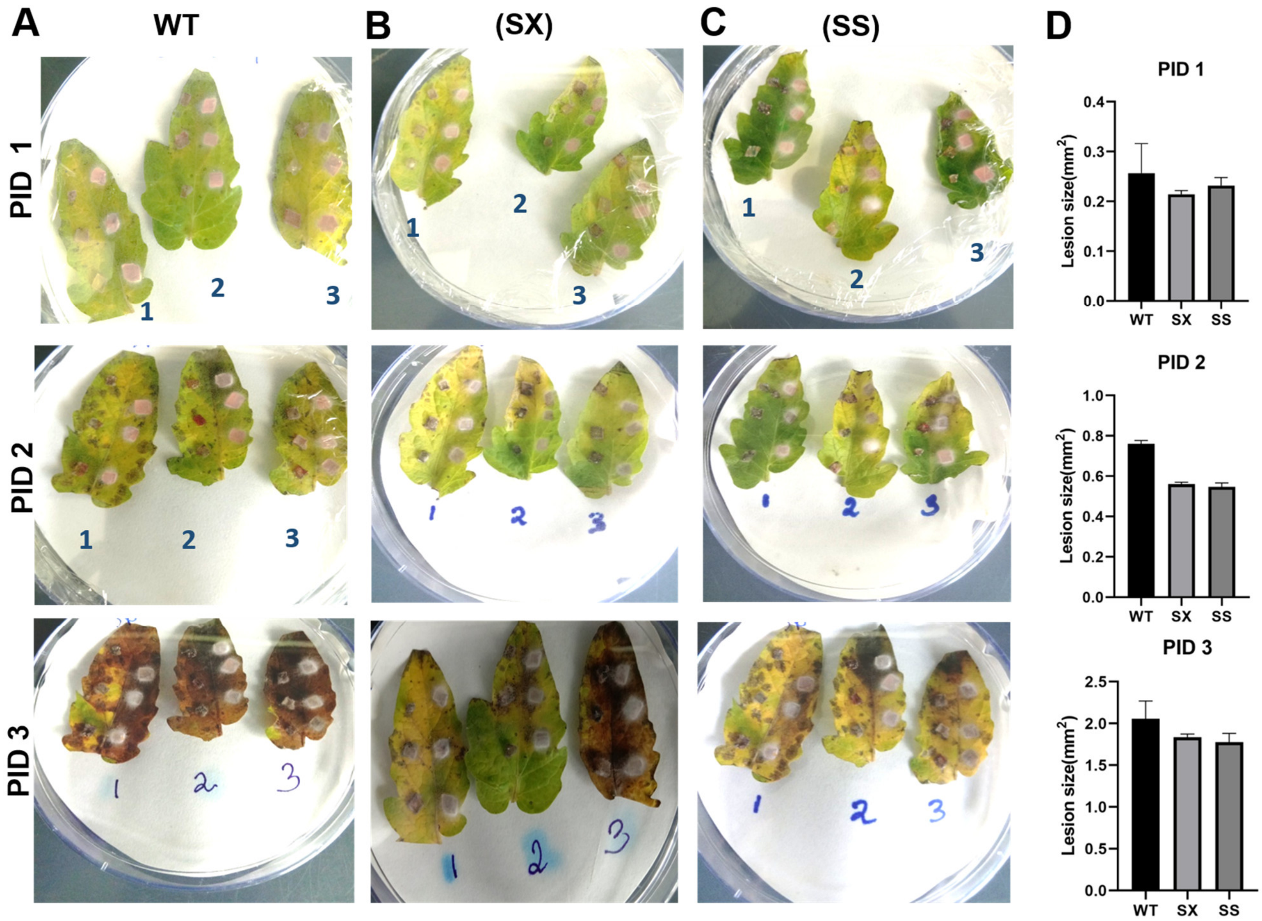

Supplementary Figure S5. Pathogen leaf disc assay for single gene CRISPR edited tomato leaves infected with *Fol* 1322 strain. Phenotypic analysis for *Fol* tolerance in single gene CRISPR edited tomato leaves of *XSP10* (SX) (**B**) and *SlSAMT* (SS) (**C**) infected with *Fol 1322* strain. WT leaves infected with *Fol 1322* strain used as control (**A**). **D**. Graphical representation of the average mean area (mm^2) of infected lesions of control wild-type (WT) and single gene CRISPR edited constructs in transient agro-infiltrated leaves of tomato. The area of lesions was measured by Image J software and the average size of lesion taken for statistical significance. Bars represent the average means ±SE of measurements from 3 lesion spots from 3 different leaf discs of control and single gene CRISPR/Cas9 constructs. The asterisk denotes a significant difference determined by t-test (P*<0.05). Single gene CRISPR editing of *XSP10* and *SlSAMT* showed subtle phenotypic response against *Fol* infection. PID: Post infection day.

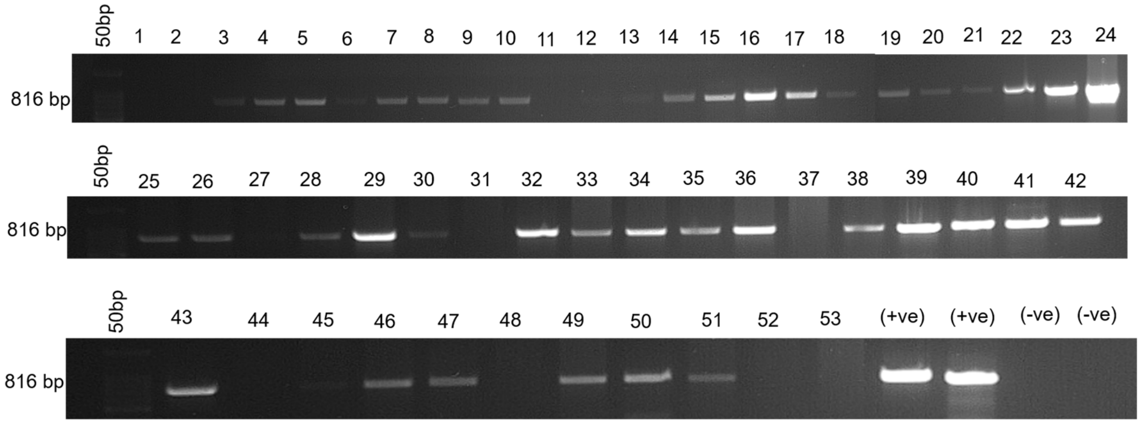

Supplementary Figure S6. Positive transformants of CRISPR/Cas9 construct at GE_0 generation in tomato cv. AV. PCR confirmation of CRISPR/Cas9 transformants using the Cas9 primers. (+ve) represents the Cas9 plasmid and untransformed genomic DNA of WT as (–ve) control.

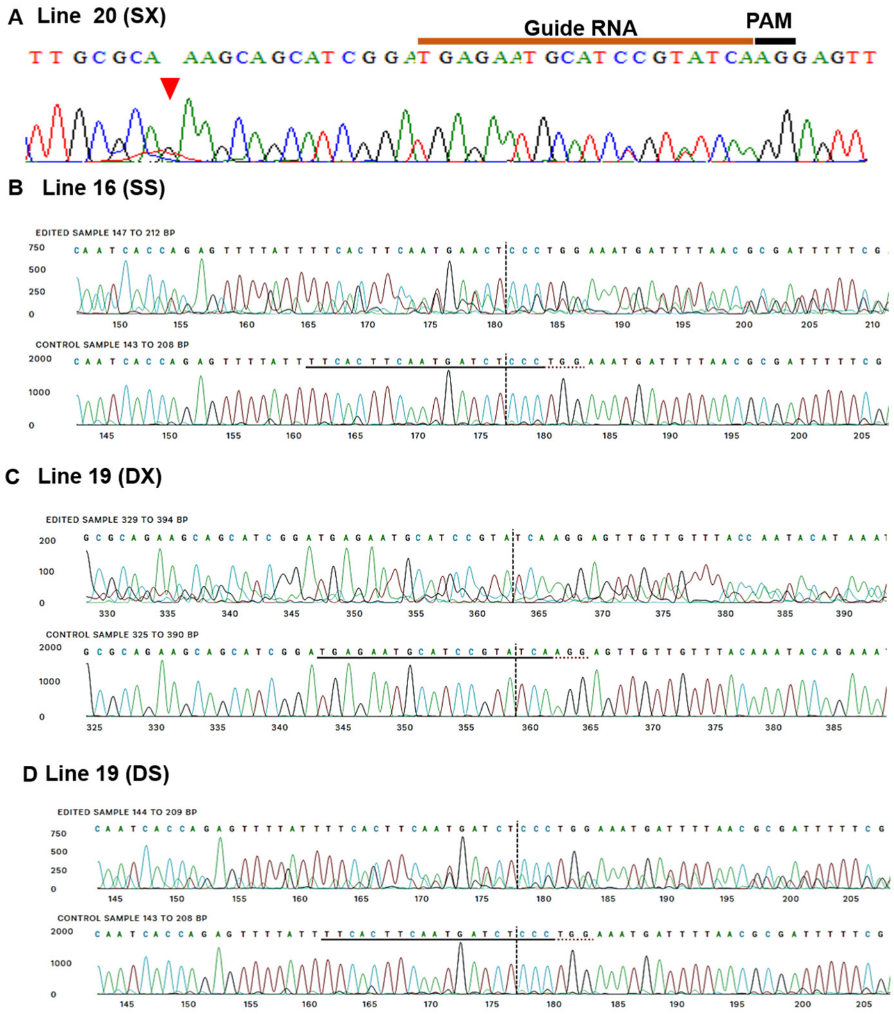

Supplementary Figure S7. Chromatograms confirming the CRISPR editing events of single and dual gene of tomato cv. AV at GE_0 generation. Dotted vertical represents cas9 cleavage sites and highlighted marks represents the guide RNA region.

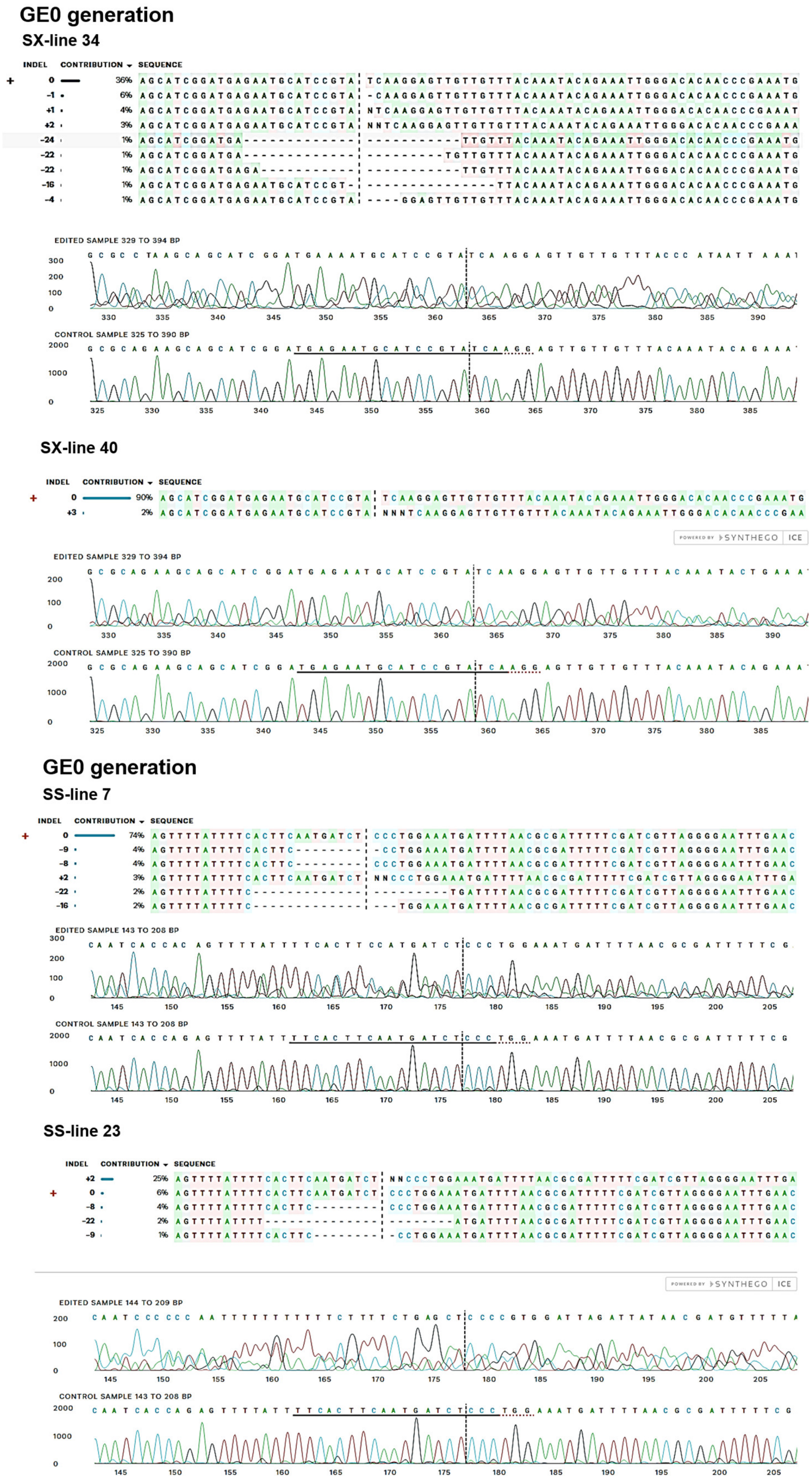
GE0 generation
SX-line 34
SX-line 40
GE0 generation
SS-line 7
SS-line 23
EDITED SAMPLE 329 TO 394 BP
CONTROL SAMPLE 325 TO 390 BP
EDITED SAMPLE 143 TO 208 BP
CONTROL SAMPLE 143 TO 208 BP
EDITED SAMPLE 144 TO 209 BP
INDEL
CONTRIBUTION
SEQUENCE
POWERED BY SYNTHEGO ICE

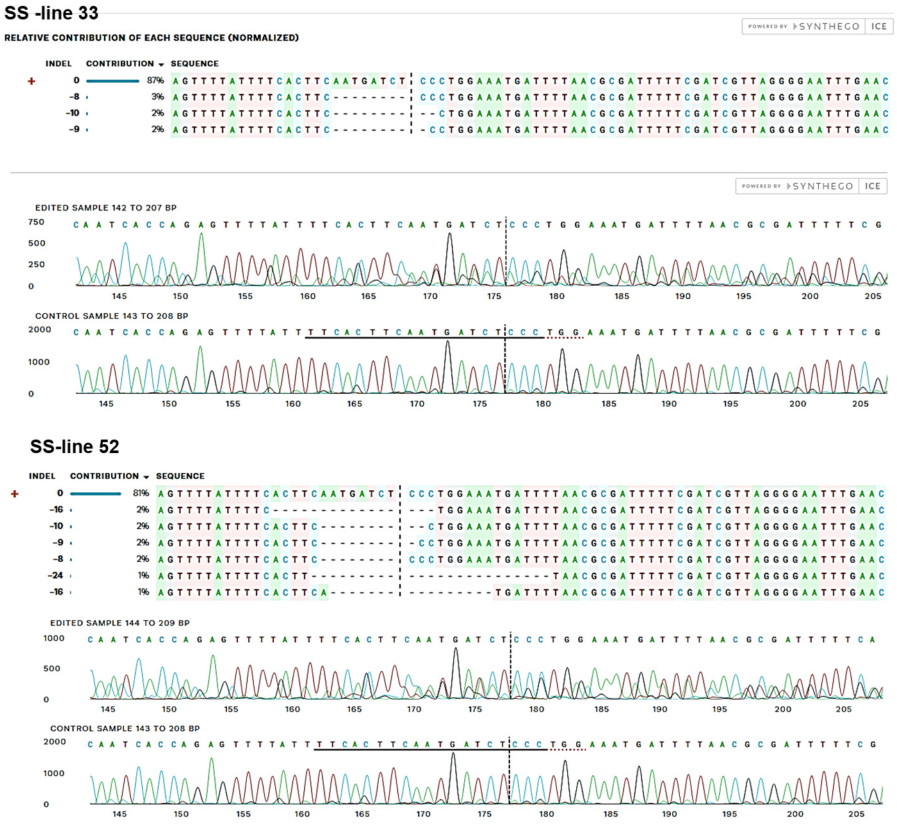

Supplementary Figure S8. Sanger sequencing and chromatogram confirming the CRISPR editing events of SX and SS of GE$_0$ generation from tomato cv. AV Dotted vertical represents cas9 cleavage sites. Dashes represents deletions of nucleotides.

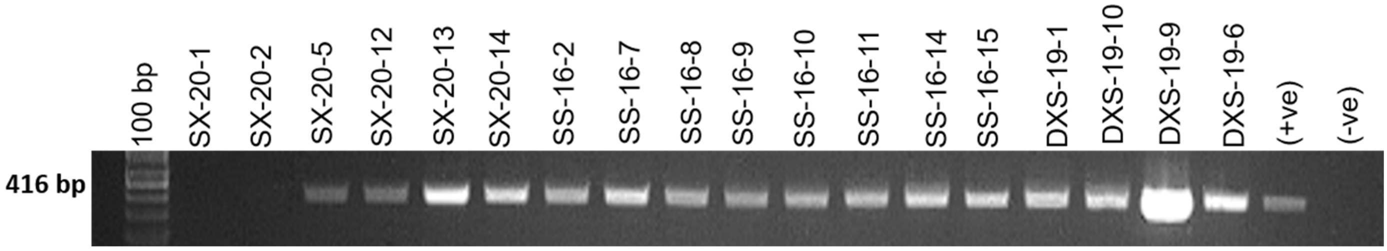

Supplementary Figure S9. Presence of T-DNA integration in GE$_1$ progeny. PCR confirmation by using sets of U6 forward primer and sgRNA scaffold reverse primer. The CRISPR plasmid taken as (+ve) control and genomic DNA of WT untransformed leaves as (-ve) control.

GE1 generation

SX -20-12

Allele1: AGCCATGAAATTGATTCCTT----CGCGCAGAAGCAGCATCGGA (insertion and deletion)
Allele2: AGCCATGAAATTGATTCCTTCGCGCCGAAGCACCATCCGA (complicated variant)
Reference: AGCCATGAAATTGATTCCTT---GCGCAGAAGCAGCATCGGATGAGAATGCATCCGTATCAAGG

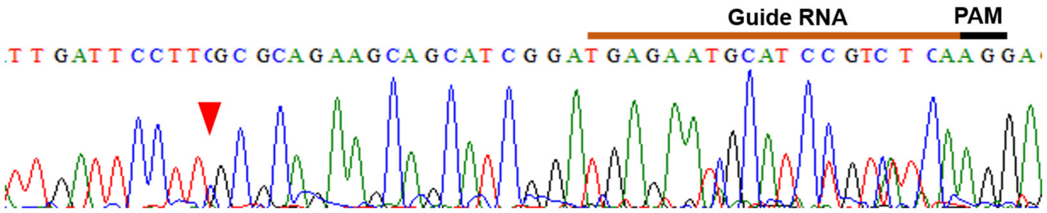

SX -20-13

Allele1: ATGAAATTGATTCCTTGCGC-GAAGCAGCATCGGAT (deletion)
Allele2: ATGAAATTGATTCCTTGCGCA-AAGCAGCATCGGAT (deletion)
Reference:ATGAAATTGATTCCTTGCGCAGAAGCAGCATCGGATGAGAATGCATCCGTTTCAAGGAG

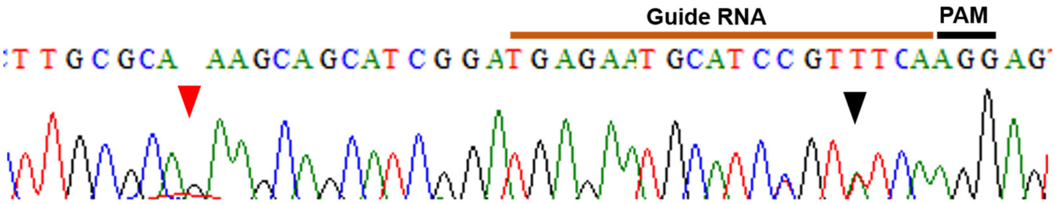

SX -20-5

Allele1: GCGCAGAAGCAGCATCGGA (WT)
Allele2: GCGCTTAAGCAGCATCGGATGAGAATGCATCCGTTTCAAGG (substitution)
Reference:GCGCAGAAGCAGCATCGGATGAGAATGCATCCGTATCAAGG

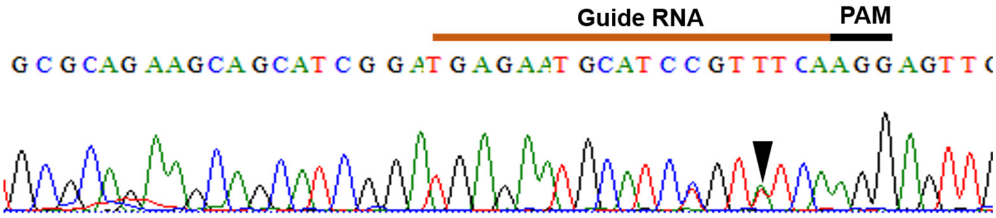

SX-20-14

Allele1: GCGCAGAAGCAGCATCGGA (WT)
Allele2: GCGCTTAAGCAGCATCGGATGAGAATGCATCTGTTTCAAGG (substitution)
Reference:GCGCAGAAGCAGCATCGGATGAGAATGCATCCGTATCAAGG

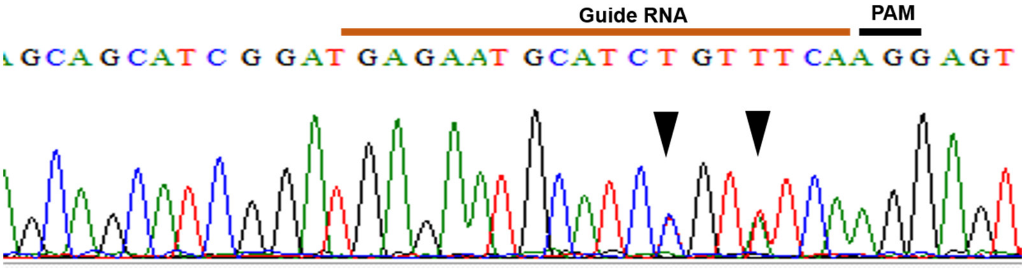

GE1 generation

SS-16-2

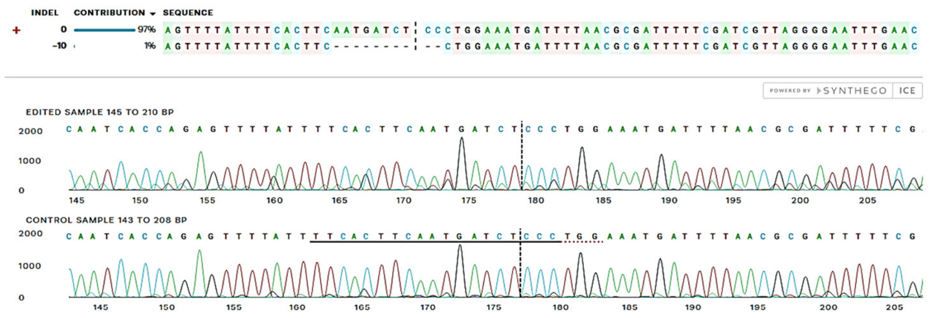

SS-16-7

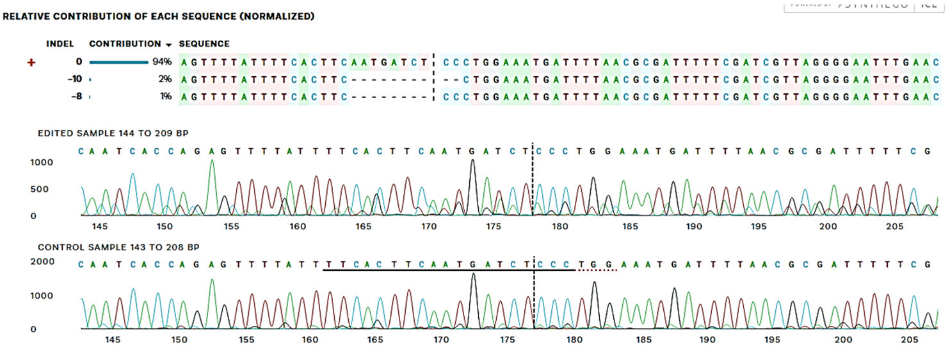

SS- 16-8

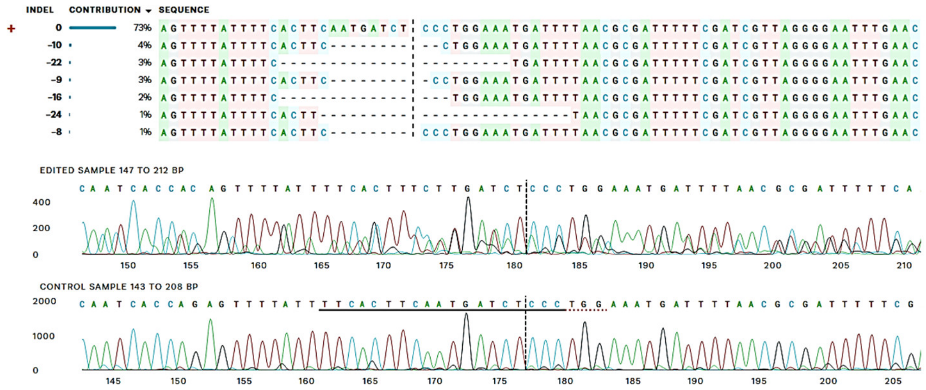

SS- 16-9

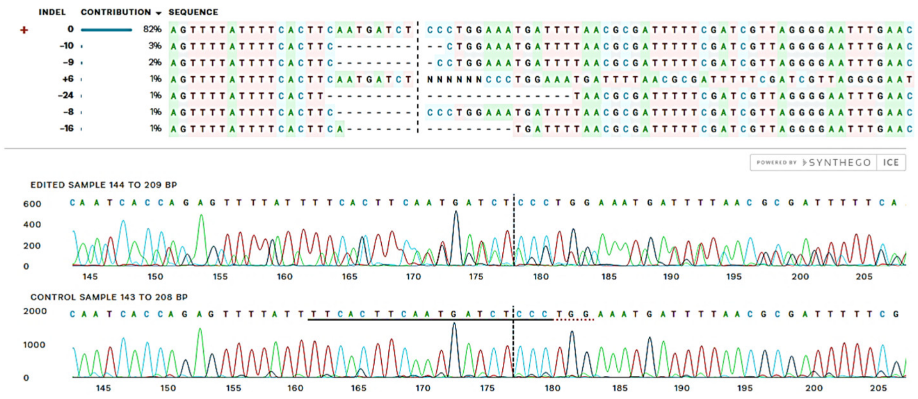

SS-16-10

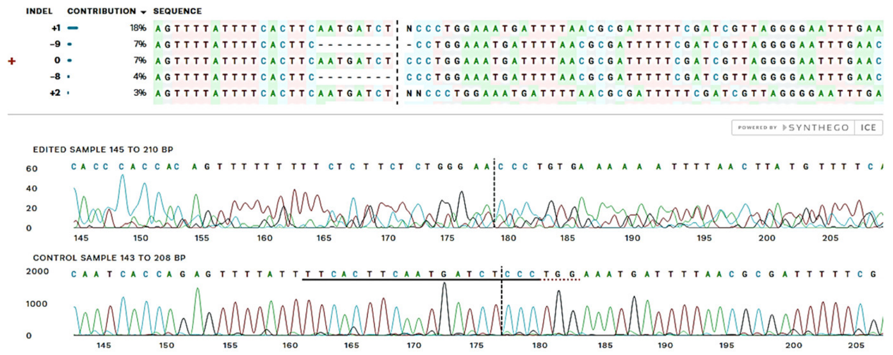

SS-16-11

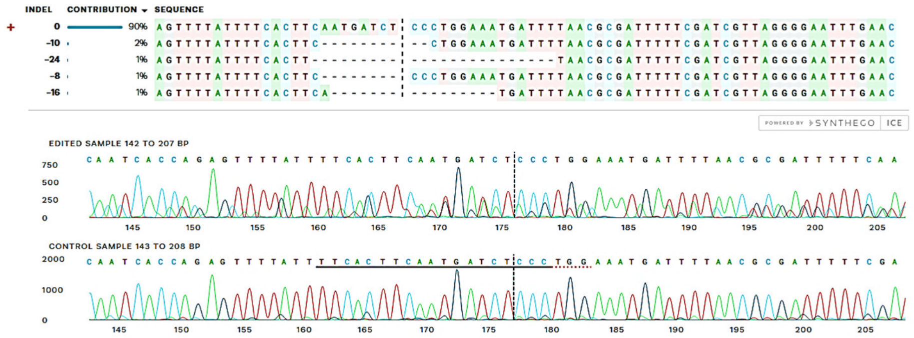

SS- 16- 14

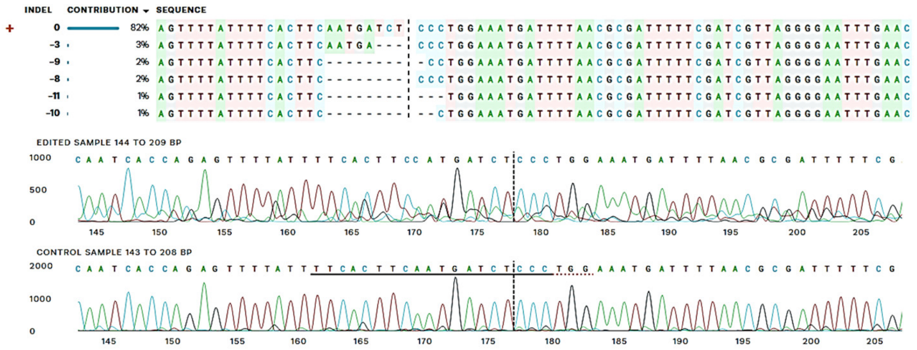

SS- 16-15

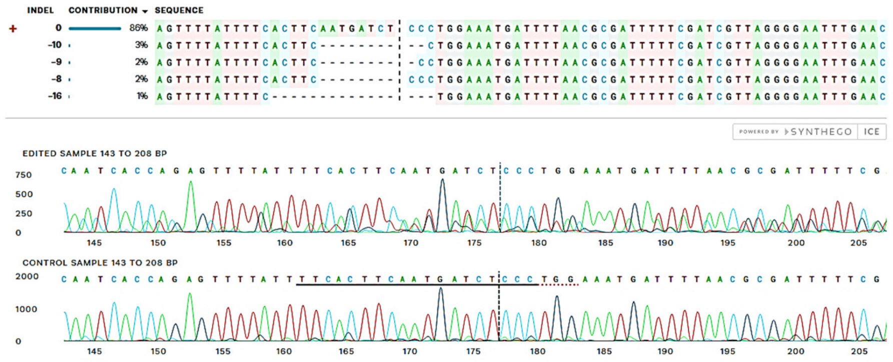

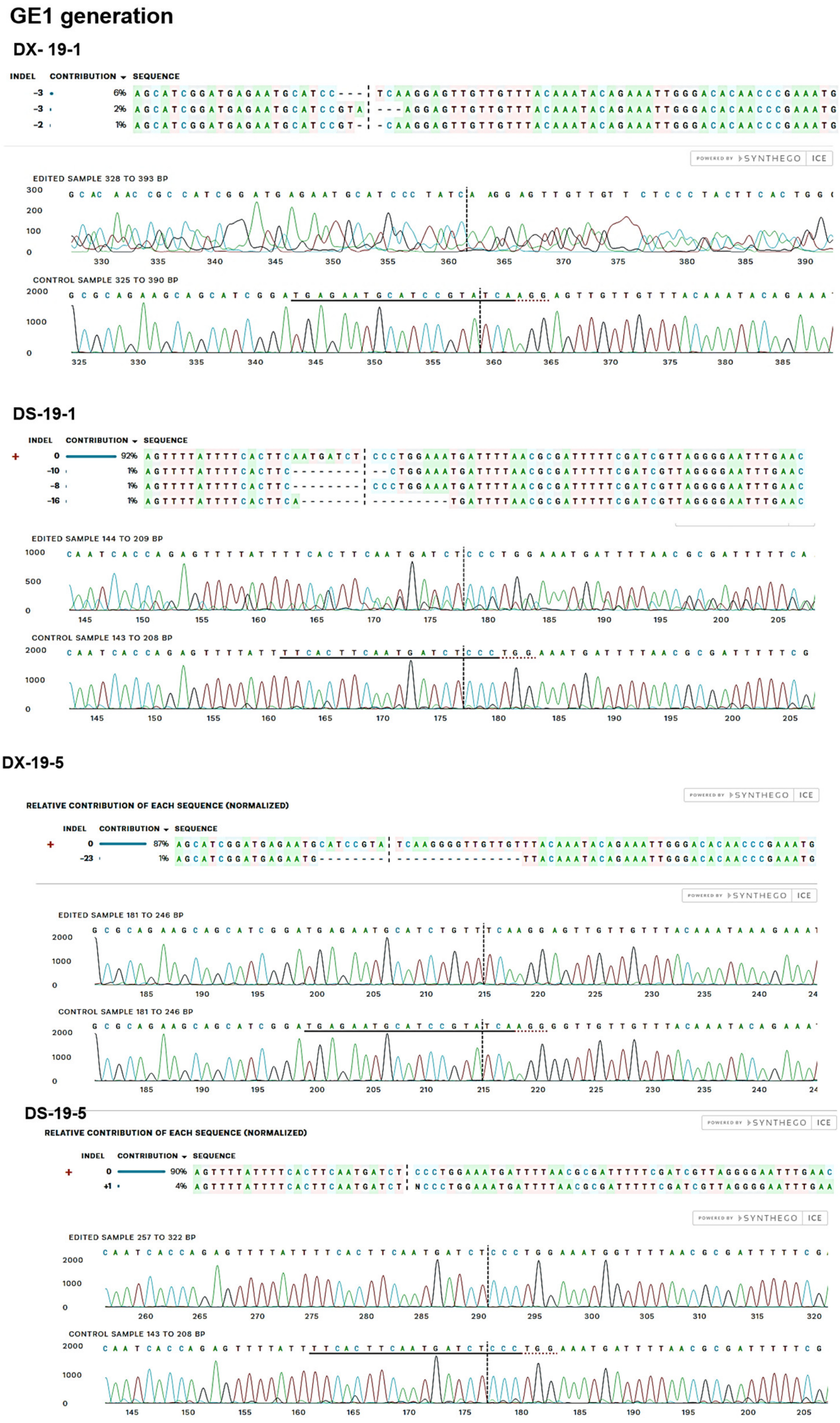
GE1 generation
DX- 19-1
INDEL
CONTRIBUTION
SEQUENCE
EDITED SAMPLE 328 TO 393 BP
CONTROL SAMPLE 325 TO 390 BP
DS-19-1
EDITED SAMPLE 144 TO 209 BP
CONTROL SAMPLE 143 TO 208 BP
DX-19-5
RELATIVE CONTRIBUTION OF EACH SEQUENCE (NORMALIZED)
EDITED SAMPLE 181 TO 246 BP
CONTROL SAMPLE 181 TO 246 BP
DS-19-5
RELATIVE CONTRIBUTION OF EACH SEQUENCE (NORMALIZED)
EDITED SAMPLE 257 TO 322 BP
CONTROL SAMPLE 143 TO 208 BP
POWERED BY SYNTHEGO ICE

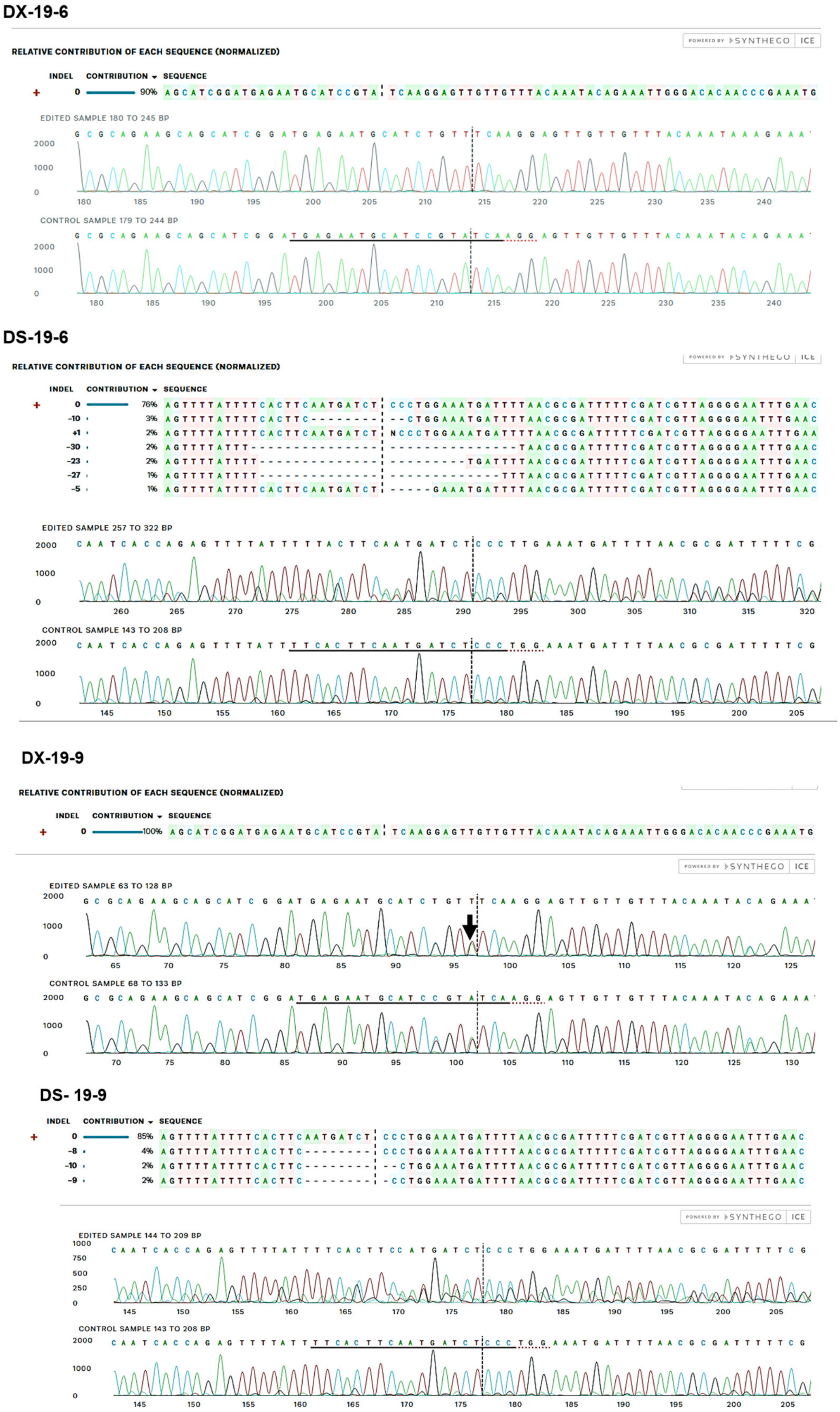
DX-19-6
RELATIVE CONTRIBUTION OF EACH SEQUENCE (NORMALIZED)
INDEL CONTRIBUTION SEQUENCE
EDITED SAMPLE 180 TO 245 BP
CONTROL SAMPLE 179 TO 244 BP
DS-19-6
RELATIVE CONTRIBUTION OF EACH SEQUENCE (NORMALIZED)
INDEL CONTRIBUTION SEQUENCE
EDITED SAMPLE 257 TO 322 BP
CONTROL SAMPLE 143 TO 208 BP
DX-19-9
RELATIVE CONTRIBUTION OF EACH SEQUENCE (NORMALIZED)
INDEL CONTRIBUTION SEQUENCE
EDITED SAMPLE 63 TO 128 BP
CONTROL SAMPLE 68 TO 133 BP
DS- 19-9
INDEL CONTRIBUTION SEQUENCE
EDITED SAMPLE 144 TO 209 BP
CONTROL SAMPLE 143 TO 208 BP

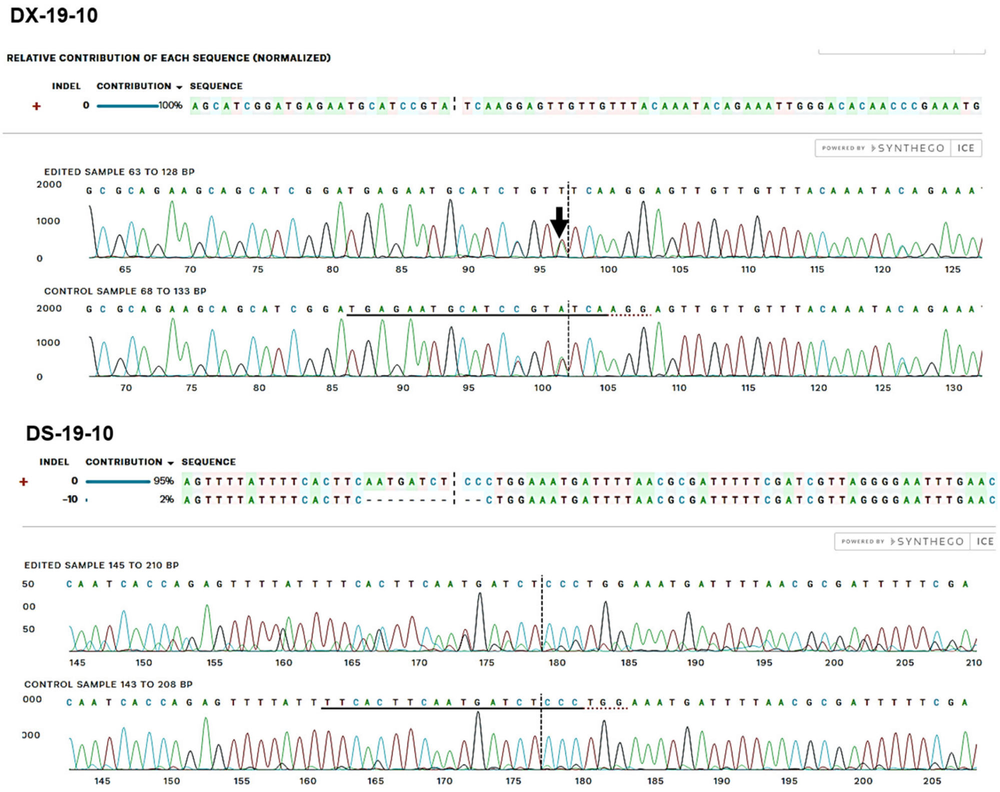

Supplementary Figure S10. Sanger sequencing and chromatogram confirming the CRISPR editing events of single (SX and SS) and dual gene (DXS) of GE$_1$ generation from tomato cv. AV. Dotted vertical represents cas9 cleavage sites. Dashes represents deletions of nucleotides.

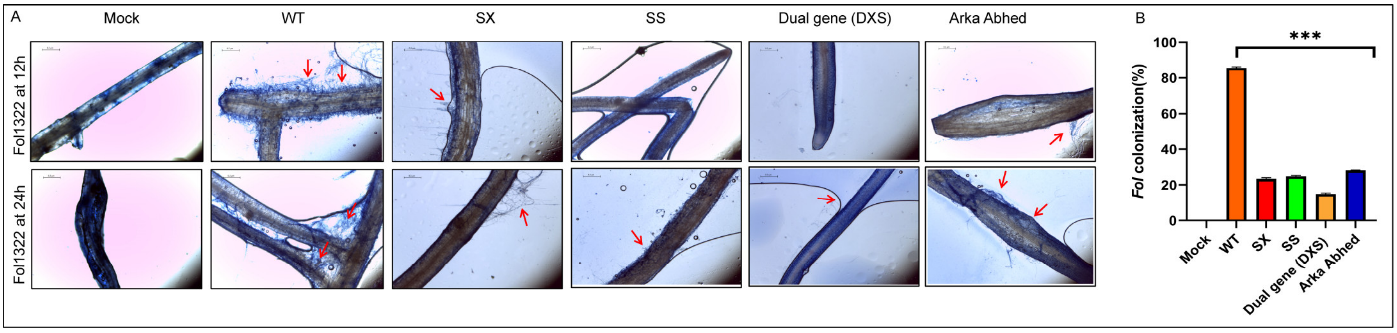

Supplementary Figure S11. ***Fol 1322* colonization in 10-12 days old seedlings of tomato cv. AV at 12 h and 24 h post-inoculation**. **A.** Phenotypic observation of WT and CRISPR edited lines roots inoculated with *Fol 1322* suspension culture. Roots treated with water (mock) were used as a control. **B.** Quantitative analysis of the intensity of root colonization by *Fol 1322* was observed at 12 h and 24 h after inoculation. The red arrow marked represents the fungal hyphae attached to the root. The colonization intensity was measured as the area of mycelia against the total size of the image. The level of significance difference (P***<0.001) was calculated by parametric student t-test using PRISM Graph Pad 9.0. Software. Scale bar 0.2 µm.

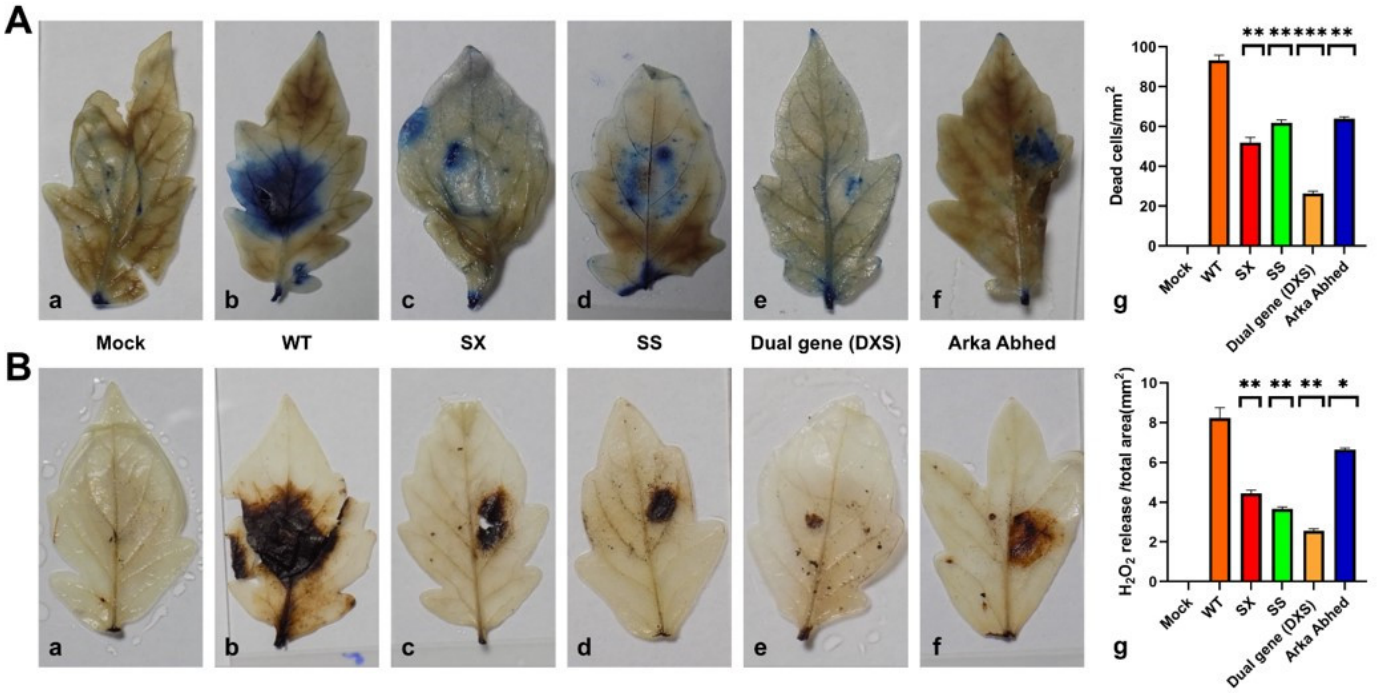

Supplementary Figure S12. Single and dual-gene CRELS of *XSP10* and *SlSAMT* showed reduced necrotic lesions upon *Fol 1322* infection in tomato leaves. A. Microscopic visualization of *Fol* infected 1 month old leaves of WT and CRELS stained with lactophenol trypan blue (a-f). Quantification of leaf cell death / mm^2 was counted with Image J software (g). **B.** DAB staining of WT and CRELS in 3-PID with *Fol 1322* (a-f). The H_2O_2 release / total area (mm^2) was measured with Image J software (g). Data were collected from three independent biological replicates, and 3 leaves were used in each biological replicate. The average lesion areas are expressed as means ± standard errors (n = 3). All the data were statistically analyzed. Asterisks indicate significant differences compared to the control (Student's t-test, *P < 0.05, **P < 0.01,***P<0.001).

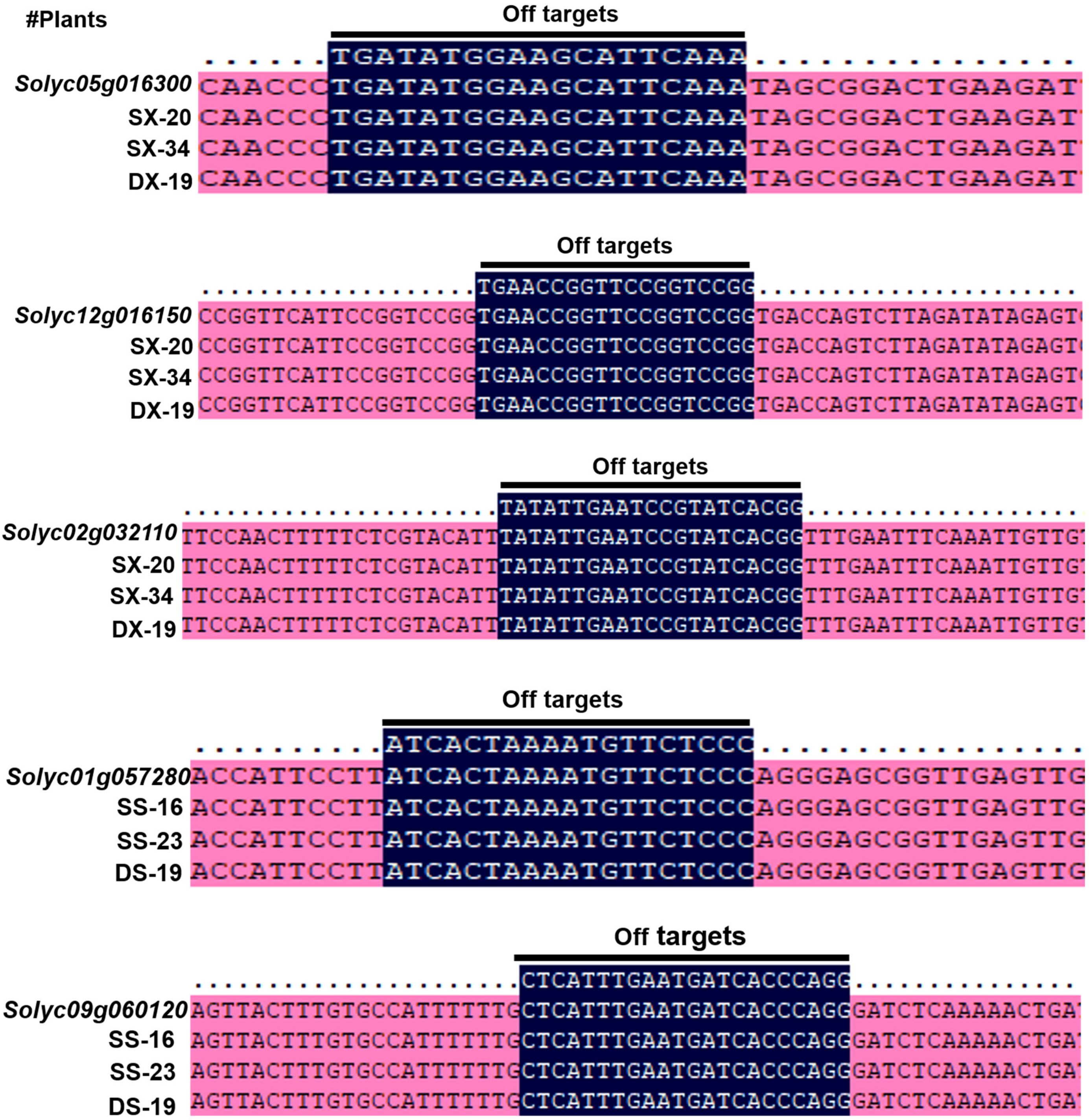

Supplementary Figure S13. Potential off-target sites generated in GE_0 CRELS by Sanger sequencing method. Sequences of CRELS (SX-20, SX-34, SS-16, SS-23, DXS-19) were taken for potential off-target analysis and aligned with reference WT by DNAMAN.10 software.

Supplementary Table S1. Primers used in this study.

Sl. No.	Gene name	Purpose	Primers (5' to 3')
1.	XSP10_PCR_Fwd	PCR	TAGGATTTGTTGTGCTACTGGC
2.	XSP10_PCR_Rev	PCR	ATGGTTATCGCAACTTCAGGAT
3.	SlSAMT_PCR_Fwd	PCR	GTGATTCTCATGACAAAGCCAA
4.	SlSAMT_PCR_Rev	PCR	TCAAATTTTGTTCAAATTCCCC
5.	M13 (-40)_ Fwd	Sequencing	GGATGTTTTCCCAGTCACGACG
6.	M13_Rev	Sequencing	GCCAGGAAACAGCTATGACC
7.	sgRNA SAMT_Fwd	CRISPR PCR	TTCACTTCAATGATCTCCC
8.	sgRNA XSP10_Fwd	CRISPR PCR	TGAGAATGCATCCGTATCA
9.	Cas9_Fwd	CRISPR PCR	CGGAACTAACTCTGTTGGATGGGC
10.	Cas9_Rev	CRISPR PCR	CCGATCTGAGCGAGGAGGTTAT
11.	Off-target_1_Fwd (*Solyc05g016300)*	CRISPR PCR	GGGCGATGAAGAAACTGAAG
12.	Off-target_1_Rev (*Solyc05g016300)*	CRISPR PCR	TCATTGTAAGCAGCCTCCAC
13.	Off-target_2_Fwd (S*olyc12g016150*)	CRISPR PCR	GTCCTGTTCCATCCCGGTCC
14.	Off-target_2_Rev (S*olyc12g016150*)	CRISPR PCR	AAATACTATTCAAATGTAGTTCG
15	Off-target_3_Fwd (*Solyc02g032110*)	CRISPR PCR	GGCAAACATTCATAACTA
16.	Off-target_3_Rev (*Solyc02g032110*)	CRISPR PCR	ACAAACTGGTTAAAATCTAG
17.	Off-target_4_Fwd (*Solyc01g057280*)	CRISPR PCR	GGGCGATGAAGAAACTGAAG
18.	Off-target_4_Rev *Solyc01g057280*)	CRISPR PCR	CCCATTGACCATGAACTGTG
19.	Off-target_5_Fwd (*Solyc09g060120*)	CRISPR PCR	GGATATAGAGTGAATTAAT
20.	Off-target_5_Rev (*Solyc09g060120*)	CRISPR PCR	AACGGGAATTTTCAAACTTCG
21.	U6 promoter_Fwd	CRISPR PCR	AGAAATCTCAAAATTCCGGCAGA
22.	sgRNA scaffold_Rev	CRISPR PCR	CCGACTCGGTGCCACTTTT

Supplementary Table S2. Summary of sgRNA design of target genes *XSP10* and *SlSAMT* along with off-target details.

Coordinates	Mismatch	sgRNA sequence	PAM	Distance		Gene name	Gene ID	Efficacy	Specificity
**On-target (*XSP10*)**									
SL2.50ch03:51687162-51687184	0	TGAGAATGCATCCGTATCA	AGG	0	Exon	Xylem sap protein 10; Protease inhibitor/seed storage/lipid transfer protein family protein (AHRD V1 **** D7LUP2_ARALY); contains Interpro domain(s) IPR003612 Plant lipid transfer protein/seed storage/trypsin-alpha amylase inhibitor	Solyc03g079880.2.1	0.66	98.83
**Off-targets**									
SL2.50ch02:27537596-27537618	4	TTATATTGAATCCGTATCA	CGG	14834	-	Unknown Protein (AHRD V1)	Solyc02g032110.1.1		
SL2.50ch12:6223854-6223876	4	TGATATTGGACCCGTATCA	CGG	9236	-	MADS-box protein (Fragment) (AHRD V1 *-*- Q84MI1_DRANE); contains Interpro domain(s) IPR002100 Transcription factor, MADS-box	Solyc12g016150.1.1		
SL2.50ch05:15474733-15474755	4	TTTGAATGCTTCCATATCA	GGG	466	-	Lysine-specific histone demethylase 1 homolog 3 (AHRD V1 ***- LDL3_ARATH)	Solyc05g016300.1.1		
**On-target (*SlSAMT*)**									
**Coordinates**	**Mismatch**	**sgRNA sequence**	**PAM**	**Distance**		**Gene name**	**Gene ID**	**Efficacy**	**Specificity**

SL2.50ch09:70803857-70803879	0	TTCACTTCAATGATCTCCC	TGG	0	Exon	Salicylic acid carboxyl methyltransferase (AHRD V1 **** C3VIX6_9ASTR); contains Interpro domain(s) IPR005299 SAM dependent carboxyl methyltransferase	Solyc09g091550.2.1	0.68	100
**Off targets**									
SL2.50ch01:60328478-60328500	4	ATCACTAAAATGTTCTCCC	AGG	1243	-	26S proteasome non-ATPase regulatory subunit 9 (AHRD V1 ***- B4FPX0_MAIZE); contains Interpro domain(s) IPR001478 PDZ/DHR/GLGF	Solyc01g057280.1.1		
SL2.50ch09:57641187-57641209	4	CTCATTTGAATGATCACCC	AGG	22079	-	Transducin family protein (AHRD V1 ***- D7KAM3_ARALY); contains Interpro domain(s) IPR011046 WD40 repeat-like	Solyc09g060120.1.1		

Supplementary Table S3. Data on measurement of lesion size upon *Fol 1322* infection in WT and single gene CRISPR edited constructs of (SX) and (SS) using Image J software.

Target gene	No. of leaves	Area	mean	stdev	Min	max
**PID 1**						
(WT)	1	0.325	100.5	10.967	55	129
	2	0.224	99.714	11.305	45	129
	3	0.222	104.11	6.251	89	129
(SX)	1	0.223	88.086	27.461	34	138
	2	0.21	77.857	21.671	39	142
	3	0.211	91.587	29.474	25	125
(SS)	1	0.226	86.903	20.357	24	126
	2	0.25	71.732	28.077	18	126
	3	0.22	77.015	26.22	18	126
**PID 2**						
(WT)	1	0.74	121.968	17.573	80	153
	2	0.77	114.909	9.677	82	140
	3	0.67	132.997	14.299	88	164
(SX)	1	0.566	88.434	13.485	39	125
	2	0.536	87.015	16.677	32	125
	3	0.552	92.545	8.623	72	122
(SS)	1	0.532	77.534	13.895	35	108
	2	0.521	77.823	17.16	20	108
	3	0.572	76.284	17.66	17	108
**PID 3**						
(WT)	1	1.912	130.845	19.283	78	165
	2	1.971	142.385	12.748	105	165
	3	2.312	139.794	11.967	107	165

(SX)	1	1.823	100.763	25.339	42	255
	2	1.811	106.5	21.914	57	239
	3	1.888	104.887	11.868	68	175
(SS)	1	1.78	82.378	36.25	20	144
	2	1.672	89.099	36.217	20	144
	3	1.88	82.144	36.978	20	144

Supplementary Table S4. Data on measurement of lesion size upon *Fol 1322* infection in WT and dual gene (DXS) constructs using Image J software**.**

	No. of Leaves	Area	Mean	StdDEV	Min	Max	XM	YM	Peri	BX	BY	Width	Height	Circ.	IntDen	%Area	Raw IntDen	AR	Round	Solidity
**PID 1**																				
	1	0.30091	98.395	12.762	72	173	360.496	1765.156	608	288	1688	144	160	0.783	2267023	0	2267023	1.111	0.9	1
	2	0.4206	96.698	22.609	40	156	920.596	1744.512	608	832	1672	160	144	0.783	2227933	0	2227933	1.111	0.9	1
**WT**	3	0.3717	95.877	26.801	38	157	1382.552	1762.171	688	1304	1672	160	184	0.782	2822620	0	2822620	1.15	0.87	1
	4	0.341	120.763	20.855	76	167	1949.673	1747.303	560	1864	1688	160	120	0.769	2318641	0	2318641	1.333	0.75	1
	5	0.312	99.904	19.356	56	146	2376.407	1827.686	704	2320	1704	120	232	0.706	2781334	0	2781334	1.933	0.517	1
	1	0.28395	67.091	14.561	31	116	2287.117	2606.119	540	2214	2544	144	126	0.782	1217295	0	1217295	1.143	0.875	1
	2	0.25877	74.206	20.989	22	159	2587.944	2581.13	600	2514	2508	156	144	0.784	1666957	0	1666957	1.083	0.923	1
**DXS**	3	1.82	68.717	13.137	35	116	2320.368	2614.904	540	2250	2550	138	132	0.785	1251741	0	1251741	1.045	0.957	1
	4	0.211	69.752	23.1	26	163	2213.057	2625.754	540	2148	2556	138	132	0.785	1270602	0	1270602	1.045	0.957	1
	5	0.231	75.238	16.451	33	168	2355.083	2722.77	540	2280	2658	144	126	0.782	1365125	0	1365125	1.143	0.875	1
**PID2**																				
	1	0.723	108.864	22.41	61	189	1713.981	2784.06	1188	1554	2646	336	258	0.772	9437171	0	9437171	1.302	0.768	1
	2	0.7967	104.967	24.73	35	187	1730.951	2194.25	1080	1602	2064	270	270	0.785	7652118	0	7652118	1	1	1
**WT**	3	0.7045	106.096	27.48	27	170	1703.101	1725.885	828	1602	1632	210	204	0.785	4545166	0	4545166	1.029	0.971	1
	4	0.712	105.809	12.263	68	170	1710.859	2743.684	780	1614	2646	198	192	0.785	4022427	0	4022427	1.031	0.97	1
	5	0.721	107.218	10.801	69	149	1692.202	2749.69	720	1596	2664	192	168	0.782	3458422	0	3458422	1.143	0.875	1
	1	0.5063	78.063	21.929	33	181	2370.585	2629.935	684	2268	2550	186	156	0.779	2265066	0	2265066	1.192	0.839	1
	2	0.552	66.742	13.893	31	116	2299.671	2611.435	588	2220	2544	156	138	0.782	1436818	0	1436818	1.13	0.885	1
**DXS**	3	0.5024	78.317	31.013	37	179	2690.507	2550.137	624	2604	2484	162	150	0.784	1903111	0	1903111	1.08	0.926	1
	4	0.542	72.264	13.595	34	121	2349.03	2621.811	552	2274	2556	144	132	0.784	1373589	0	1373589	1.091	0.917	1
	5	0.412	68.037	13.161	33	116	2322.995	2634.563	468	2262	2580	120	114	0.785	930742	0	930742	1.053	0.95	1
**PID3**																				
	1	1.23	68.518	25.593	16	174	1566.832	2617.009	1380	1332	2508	480	210	0.665	6906645	0	6906645	2.286	0.438	1
	2	1.2967	115.292	33.031	46	162	1640.225	2291.915	480	1584	2238	120	120	0.785	1660206	0	1660206	1	1	1
**WT**	3	1.2045	100.018	14.423	53	141	1686.527	1614.504	492	1620	1560	132	114	0.781	1505065	0	1505065	1.158	0.864	1
	4	1.112	128.784	18.675	74	169	1631.548	1271.504	432	1584	1212	96	120	0.776	1483593	0	1483593	1.25	0.8	1
	5	1.221	146.913	27.546	82	203	1527.593	556.763	552	1458	498	150	126	0.779	2776656	0	2776656	1.19	0.84	1
	1	0.4063	80.882	24.672	32	154	1864.787	2724.063	876	1740	2616	228	210	0.784	3872637	0	3872637	1.086	0.921	1
	2	0.452	79.302	30.207	14	182	1891.588	2728.946	1416	1680	2562	408	300	0.767	9706561	0	9706561	1.36	0.735	1
**DXS**	3	0.3024	76.989	24.627	29	154	1854.833	2714.572	948	1722	2592	240	234	0.785	4323711	0	4323711	1.026	0.975	1
	4	0.412	82.45	17.246	32	147	1979.214	1580.282	744	1878	1494	198	174	0.782	2840563	0	2840563	1.138	0.879	1
	5	0.4212	66.978	21.441	26	148	1812.105	2670.859	660	1722	2580	162	168	0.785	1822865	0	1822865	1.037	0.964	1
**PID4**																				
	1	1.432	130.59	17.968	82	206	1792.989	349.374	444	1740	294	108	114	0.785	2037992	0	2037992	1.056	0.947	1
	2	1.4967	166.57	13.575	110	213	1775.427	352.752	408	1722	306	108	96	0.783	1782159	0	1782159	1.125	0.889	1
**WT**	3	1.4045	169.58	11.439	142	207	1778.775	353.128	348	1734	312	90	84	0.784	1296440	0	1296440	1.071	0.933	1
	4	1.321	170.93	15.581	91	213	1780.947	349.685	444	1722	300	120	102	0.78	2090719	0	2090719	1.176	0.85	1
	5	1.422	162.156	25.686	79	213	1788.333	356.419	516	1722	300	138	120	0.782	2653783	0	2653783	1.15	0.87	1
	1	0.5063	51.421	11.433	52	130	1825.599	2376.727	504	1758	2316	132	120	0.784	1444144	0	1444144	1.1	0.909	1
	2	0.552	47.137	25	19	172	2045.225	1591.524	804	1938	1506	222	180	0.777	3325339	0	3325339	1.233	0.811	1
**DXS**	3	0.5024	53.614	15.192	25	144	1788.872	1545.373	396	1740	1500	102	96	0.785	779997	0	779997	1.062	0.941	1
	4	0.437	56.57	12.561	59	127	1917.289	1668.014	456	1854	1614	126	102	0.777	1173468	0	1173468	1.235	0.81	1
	5	0.469	54.615	13.527	40	162	1722.728	1549.828	540	1650	1488	144	126	0.782	1512409	0	1512409	1.143	0.875	1

t-test: Paired Two Sample for Means	PID3	
	Variable 1	*Variable 2*
Mean	159.9798	108.8724
Variance	281.3738617	1035.933612
Observations	5	5
Pearson Correlation	0.523200526	
Hypothesized Mean Difference	0	
df	4	
t Stat	4.166344386	
P(T<=t) one-tail	0.007036365	
t Critical one-tail	2.131846786	
P(T<=t) two-tail	0.01407273	**
t Critical two-tail	2.776445105	

t-test: Paired Two Sample for Means	PID 4	
	Variable 1	*Variable 2*
	68.518	*80.882*
Mean	122.75175	76.42975
Variance	397.5417669	44.71385625
Observations	4	4
Pearson Correlation	-0.56551063	
Hypothesized Mean Difference	0	
df	3	
t Stat	3.804274871	
P(T<=t) one-tail	0.015955888	
t Critical one-tail	2.353363435	
P(T<=t) two-tail	0.0031911775	***
t Critical two-tail	3.182446305	

Supplementary Table S5. Statistical data analysis of root colonization in 10-12 days old seedlings of tomato cultivar Arka Vikas using Image J software.

WT						
		Area	Mean	StDEV	Min	Max
Total area	1	8940	208.138	66.178	15	255
	2	7980	204.184	57.825	43	253
	3	4960	207.486	55.978	43	252
Fol infected area	1	360	120.639	61.234	45	249

	2	528	121.619	55.678	45	248
	3	768	118.448	40.065	33	250
(SX)						
Total area	1	6970	63.705	27.086	27	237
	2	4550	66.129	28.449	17	238
	3	2726	64.039	30.306	27	237
Fol infected area	1	180	42.978	10.011	14	58
	2	240	41.783	12.242	11	93
	3	286	39.99	8.95	11	57
(SS)						
Total area	1	26904	88.515	77.587	12	237
	2	7812	97.049	70.978	15	232
	3	9844	99.55	51.497	25	249
Fol infected area	1	1890	67.252	23.064	11	201
	2	1680	71.443	20.283	11	163
	3	1927	74.171	44.149	11	233
Dual gene (DXS)						
Total area	1	4020	86.213	62.325	14	240
	2	4875	85.953	63.452	14	240
	3	3658	72.629	48.976	14	240
Fol infected area	1	1638	67.854	31.036	14	237
	2	2254	67.633	20.679	14	192
	3	1845	66.353	45.967	11	235
Arka Abhed						
Total area	1	1440	197.887	35.907	14	217
	2	1360	209.918	8.944	135	222
	3	1740	195.155	60.906	17	221
Fol infected area	1	812	173.878	64.973	16	235
	2	580	175.384	69.903	16	235
	3	558	177.715	56.761	30	234

**Tested lines**	***Fol* colonized (%)**	***Fol* not detected (%)**
Arka Abhed	25.33	74.67
Mock	0	100
WT	86.36	13.64
SX	23.13	76.87
SS	24.08	75.92
Dual gene (DXS)	14.31	85.69

**Table Analyzed**	**Data 1**	**Table Analyzed**	**Data 2**
Column D	**SX**	Column E	**SS**
vs.	vs.	vs.	vs.
Column C	**WT**	Column C	**WT**
Paired t test		Paired t test	
P value	0.001	P value	0.001
P value summary	***	P value summary	***
Significantly different (P < 0.05)?	Yes	Significantly different (P < 0.05)?	Yes
One- or two-tailed P value?	Two-tailed	One- or two-tailed P value?	Two-tailed
t, df	t=56.38, df=2	t, df	t=43.69, df=2
Number of pairs	3	Number of pairs	3
How big is the difference?		How big is the difference?	
Mean of differences (D - C)	-62.06	Mean of differences (E - C)	-60.5
SD of differences	1.907	SD of differences	2.398
SEM of differences	1.101	SEM of differences	1.385
95% confidence interval	-66.80 to -57.32	95% confidence interval	-66.45 to -54.54
R squared (partial eta squared)	0.9994	R squared (partial eta squared)	0.999

**Table Analyzed**	**Data 3**	**Table Analyzed**	**Data 4**
Column F	**Dual gene (DXS)**	Column G	**Arka Abhed**
vs.	vs.	vs.	vs.
Column C	**WT**	Column C	**WT**
Paired t test		Paired t test	
P value	0.001	P value	0.001
P value summary	***	P value summary	***
Significantly different (P < 0.05)?	Yes	Significantly different (P < 0.05)?	Yes
One- or two-tailed P value?	Two-tailed	One- or two-tailed P value?	Two-tailed
t, df	t=94.38, df=2	t, df	t=68.21, df=2
Number of pairs	3	Number of pairs	3
How big is the difference?		How big is the difference?	
Mean of differences (F - C)	-71.27	Mean of differences (G - C)	-60.68
SD of differences	1.308	SD of differences	1.541
SEM of differences	0.7551	SEM of differences	0.8895
95% confidence interval	-74.52 to -68.02	95% confidence interval	-64.50 to -56.85
R squared (partial eta squared)	0.9998	R squared (partial eta squared)	0.9996

Supplementary Table S6. Data measurement of cell death/mm² in infected tomato leaves upon *Fol 1322* infection in WT, single (SX and SS) and dual gene (DXS) constructs using Image J software.

Target gene		Area	StDev	Max	Min
WT	1	95.981	36.25	15	221
	2	90.822	37.648	15	221
	3	92.724	37.569	15	222
SX	1	54.396	31.236	2	209
	2	49.272	24.799	2	123
	3	52.291	27.9	9	167
SS	1	61.178	33.219	6	167
	2	60.774	33.082	6	167
	3	63.492	34.873	6	167
Dual gene (DXS)	1	25.522	10.085	3	69
	2	25.594	11.21	3	78
	3	27.641	9.143	3	61
Arka Abhed	1	64.716	29.877	2	131
	2	66.924	24.907	4	121
	3	62.875	18.51	10	103

Table Analyzed	Data 1	Table Analyzed	Data 2
Column C	**SX**	Column D	**SS**
vs.	vs.	vs.	vs.
Column B	**WT**	Column B	**WT**
Paired t test		Paired t test	
P value	0.001	P value	0.0031
P value summary	**	P value summary	**
Significantly different (P < 0.05)?	Yes	Significantly different (P < 0.05)?	Yes
One- or two-tailed P value?	Two-tailed	One- or two-tailed P value?	Two-tailed
t, df	t=108.9, df=2	t, df	t=18.05, df=2
Number of pairs	3	Number of pairs	3
How big is the difference?		How big is the difference?	
Mean of differences (C - B)	-41.19	Mean of differences (D - B)	-31.36
SD of differences	0.6552	SD of differences	3.009
SEM of differences	0.3783	SEM of differences	1.737
95% confidence interval	-42.82 to -39.56	95% confidence interval	-38.83 to -23.89
R squared (partial eta squared)	0.9998	R squared (partial eta squared)	0.9939

Table Analyzed	Data 3	Table Analyzed	Data 4
Column F	Arka Abhed	Column E	Dual gene (DXS)
vs.	vs.	vs.	vs.
Column B	WT	Column B	WT
Paired t test		Paired t test	
P value	0.0051	P value	0.0007
P value summary	**	P value summary	***
Significantly different (P < 0.05)?	Yes	Significantly different (P < 0.05)?	Yes
One- or two-tailed P value?	Two-tailed	One- or two-tailed P value?	Two-tailed
t, df	t=13.79, df=2	t, df	t=37.85, df=2
Number of pairs	3	Number of pairs	3
How big is the difference?		How big is the difference?	
Mean of differences (F - B)	-35	Mean of differences (E - B)	-66.92
SD of differences	4.398	SD of differences	3.063
SEM of differences	2.539	SEM of differences	1.768
95% confidence interval	-45.93 to -24.08	95% confidence interval	-74.53 to -59.31
R squared (partial eta squared)	0.9896	R squared (partial eta squared)	0.9986

Supplementary Table S7. Data measurement of H_2O_2 release/ mm² in infected tomato leaves upon *Fol 1322* infection in WT, single (SX and SS) and dual gene (DXS) constructs using Image J software.

		Area	Stdev	Min	Max
WT	1	8.621	50.446	3	224
	2	8.432	47.731	3	224
	3	7.63	48.371	3	224
SX	5	4.321	27.591	1	167
	6	4.62	30.629	1	192
	7	4.43	29.511	1	192
SS	1	3.765	22.944	1	192
	2	3.57	21.913	1	161
	3	3.66	22.97	1	192
Dual gene (DXS)	1	2.543	14.77	1	117
	2	2.671	15.832	1	144
	3	2.45	15.907	1	128
Arka Abhed	1	6.721	46.018	1	197
	2	6.56	42.823	1	208
	3	6.661	38.385	1	208

Table Analyzed	**Data 1**	Table Analyzed	**Data 2**
Column C	**SX**	Column D	**SS**
vs.	vs.	vs.	vs.
Column B	**WT**	Column B	**WT**
Paired t test		Paired t test	
P value	0.0029	P value	0.0014
P value summary	**	P value summary	**
Significantly different (P < 0.05)?	Yes	Significantly different (P < 0.05)?	Yes
One- or two-tailed P value?	Two-tailed	One- or two-tailed P value?	Two-tailed
t, df	t=18.43, df=2	t, df	t=26.96, df=2
Number of pairs	3	Number of pairs	3
How big is the difference?		How big is the difference?	
Mean of differences (C - B)	-41.97	Mean of differences (D - B)	-49.42
SD of differences	3.944	SD of differences	3.175
SEM of differences	2.277	SEM of differences	1.833
95% confidence interval	-51.76 to -32.17	95% confidence interval	-57.31 to -41.53
R squared (partial eta squared)	0.9941	R squared (partial eta squared)	0.9973

Table Analyzed	**Data 3**	Table Analyzed	**Data 4**
Column F	**Arka Abhed**	Column E	**Dual gene (DXS)**
vs.	vs.	vs.	vs.
Column B	**WT**	Column B	**WT**
Paired t test		Paired t test	

P value	0.01	P value	0.001
P value summary	*	P value summary	**
Significantly different (P < 0.05)?	Yes	Significantly different (P < 0.05)?	Yes
One- or two-tailed P value?	Two-tailed	One- or two-tailed P value?	Two-tailed
t, df	t=9.917, df=2	t, df	t=30.96, df=2
Number of pairs	3	Number of pairs	3
How big is the difference?		How big is the difference?	
Mean of differences (F - B)	-25.15	Mean of differences (E - B)	-56.02
SD of differences	4.393	SD of differences	3.134
SEM of differences	2.536	SEM of differences	1.81
95% confidence interval	-36.07 to -14.24	95% confidence interval	-63.80 to -48.23
R squared (partial eta squared)	0.9801	R squared (partial eta squared)	0.9979

Supplementary Table S8. Data measurement of fresh weight (g) of Mock, WT, single genes (SX and SS), dual gene (DXS) and Arka Abhed upon *Fol 1322* infected tomato plants.

**Fresh weight (g)**					
Mock	WT	SX	SS	Dual gene DXS	Arka Abhed
25.5	9.92	12.32	16.21	20.18	14.22
28.65	7.35	15.21	12.21	18.42	12.44
24.66	7.67	16.7	10.33	18.13	12.41
26.44	8.86	12.31	12.31	17.46	11.43

**Table Analyzed**	**Data 1**	**Table Analyzed**	**Data 2**
Column C	**SX**	Column D	**SS**
vs.	vs.	vs.	vs.
Column B	**WT**	Column B	**WT**
Paired t test		Paired t test	
P value	0.0396	P value	0.0125
P value summary	*	P value summary	*
Significantly different (P < 0.05)?	Yes	Significantly different (P < 0.05)?	Yes
One- or two-tailed P value?	Two-tailed	One- or two-tailed P value?	Two-tailed
t, df	t=3.497, df=3	t, df	t=5.392, df=3
Number of pairs	4	Number of pairs	4
How big is the difference?		How big is the difference?	
Mean of differences (C - B)	5.685	Mean of differences (D - B)	4.315

SD of differences	3.251	SD of differences	1.601
SEM of differences	1.625	SEM of differences	0.8003
95% confidence interval	0.5120 to 10.86	95% confidence interval	1.768 to 6.862
R squared (partial eta squared)	0.803	R squared (partial eta squared)	0.9065

Table Analyzed	**Data 3**	**Table Analyzed**	**Data 4**
Column E	**Dual gene (DXS)**	Column F	**Arka Abhed**
vs.	vs.	vs.	vs.
Column B	**WT**	Column B	**WT**
Paired t test		Paired t test	
P value	0.0003	P value	0.04
P value summary	***	P value summary	*
Significantly different (P < 0.05)?	Yes	Significantly different (P < 0.05)?	Yes
One- or two-tailed P value?	Two-tailed	One- or two-tailed P value?	Two-tailed
t, df	t=19.12, df=3	t, df	t=7.470, df=3
Number of pairs	4	Number of pairs	4
How big is the difference?		How big is the difference?	
Mean of differences (E - B)	10.1	Mean of differences (F - B)	4.175
SD of differences	1.056	SD of differences	1.118
SEM of differences	0.5281	SEM of differences	0.5589
95% confidence interval	8.417 to 11.78	95% confidence interval	2.396 to 5.954
R squared (partial eta squared)	0.9919	R squared (partial eta squared)	0.949

Supplementary Table S9. Data measurement of disease severity of single and dual gene (DXS) in 4 weeks old tomato infected plants.

**Disease index (%)**	**0**	**1**	**2**	**3**	**4**
Mock	100				
WT			25		75
SX		25	25	50	
Dual gene (DXS)		25	25	25	25
Dual gene DXS	75			25	
Arka Abhed		25	25	50	

Table Analyzed	Data 1
Kruskal-Wallis test	
P value	0.0465
Exact or approximate P value?	Exact
P value summary	*
Do the medians vary signif. (P < 0.05)?	Yes
Number of groups	5
Kruskal-Wallis statistic	8.562
Data summary	
Number of treatments (columns)	5
Number of values (total)	15

Supplementary Table S10. Data measurement of fungus colonization in WT and CRELs upon *Fol 1322* infection in tomato cv. AV

	**No fungus**	**Cotyledon node**	**2nd node**	**4th node**	**Total sliced stem**
**Mock**	0				12
**WT**		4	3	3	12
**SX**		3	3		12
**SS**		2	2		12
**Dual gene (DXS)**		2	1		12
**Arka Abhed**		3	3	1	12

**Fungus colonization (%)**	**No fungus**	**Cotyledon node**	**2nd node**	**4th node**
**Mock**	0			
**WT**	16.67	33.33	25	25
**SX**	50	25	25	
**SS**	66.68	16.66	16.66	
**Dual gene (DXS)**	75.01	16.66	8.33	
**Arka Abhed**	41.67	25	25	8.33

**Table Analyzed**	Data 1				
Two-way ANOVA	Ordinary				
Alpha	0.05				
Source of Variation	% of total variation	P value	P value summary	Significant?	
Row Factor	31.17	0.1365	ns	No	
Column Factor	27.93	0.0659	ns	No	
ANOVA table	SS (Type III)	DF	MS	F (DFn, DFd)	P value
Row Factor	2654	5	530.9	F (5, 12) = 2.095	P=0.1365
Column Factor	2378	3	792.6	F (3, 12) = 3.127	P=0.0659
Residual	3041	12	253.4		
Data summary					
Number of columns (Column Factor)	4				
Number of rows (Row Factor)	6				
